# The probiotic *Limosilactobacillus fermentum* CECT5716 enhances the antihypertensive response to hydrochlorothiazide in spontaneously hypertensive rats

**DOI:** 10.1080/19490976.2025.2586324

**Published:** 2025-11-18

**Authors:** Cristina González-Correa, Sofia Miñano, Javier Moleón, Marta Toral, Iñaki Robles-Vera, Manuel Sánchez, Rosario Jiménez, Monica Olivares, Natividad Martín-Morales, Francisco O'Valle, Eduardo Guerra-Hernández, Miguel Romero, Manuel Gómez-Guzmán, Juan Duarte

**Affiliations:** a Department of Pharmacology, School of Pharmacy and Center for Biomedical Research (CIBM), University of Granada, Granada, Spain; b Instituto de Investigación Biosanitaria de Granada, ibs.GRANADA, Granada, Spain; c Department of Translational Rheumatology and Immunology, Institute of Musculoskeletal Medicine, University of Münster, Münster, Germany; d Ciber de Enfermedades Cardiovasculares (CIBERCV), Madrid, Spain; e Centro Nacional de Investigaciones Cardiovasculares (CNIC), Madrid, Spain; f Biosearch S.A.U. (a Kerry® Company), R&D Department, Granada, Spain; g Instituto de Biopatología y Medicina Regenerativa (IBIMER), Department of Pathology, School of Medicine, University of Granada, Granada, Spain; h Department of Nutrition and Bromatology, University of Granada, Granada, Spain

**Keywords:** Hypertension, fecal transplant, gut dysbiosis, immune system, *Limosilactobacillus*, hydrochlorothiazide

## Abstract

*Limosilactobacillus fermentum* CECT5716 (LC40) consumption reduces hypertension and improves endothelial dysfunction in spontaneously hypertensive rats (SHRs). The diuretic hydrochlorothiazide (HCTZ) lowers blood pressure in SHR but disrupts the gut microbiota balance. In this study, we investigated whether the LC40 could enhance the antihypertensive effects of HCTZ. Interestingly, we found that coadministration of LC40 with HCTZ potentiated the beneficial effects of HCTZ on endothelial dysfunction and blood pressure without altering plasma HCTZ concentrations or exacerbating electrolyte imbalances. These protective effects were associated with normalization of microbiota alterations, including a reduction in the Firmicutes/Bacteroidota ratio, suppression of lipopolysaccharide biosynthesis, and an increase in acetate-producing bacteria. Additionally, LC40 reduced intestinal pathology and endotoxemia. Furthermore, the HCTZ + LC40-treated rats exhibited reduced neuroinflammation and sympathetic activity, along with an immunoregulatory effect characterized by increased regulatory T cell infiltration and a reduction of vascular oxidative stress in the aorta. The beneficial effects of LC40 in HCTZ-treated rats appeared to be microbiota dependent, as they were replicated through fecal microbiota transplantation in germ-depleted normotensive rats. Our findings identify the gut microbiota as a novel therapeutic target to enhance the antihypertensive effects of diuretics. The coadministration of LC40 with HCTZ modulates immune responses, providing a promising strategy to improve hypertension management.

## Introduction

Hypertension is the primary risk factor for cardiovascular disease and the leading cause of death worldwide.^1^ Between 1996 and 2021, angiotensin-converting enzyme (ACE) inhibitors and thiazides remained the most prescribed classes of antihypertensive medications in the United States, with hydrochlorothiazide (HCTZ) being the most prevalent thiazide diuretic, accounting for 53% of prescriptions.^2^ Despite the availability of multiple antihypertensive medications, 10% to 30% of hypertensive patients continue to exhibit blood pressure levels above 130/90 mmHg despite receiving three antihypertensive drugs, a condition known as resistant hypertension. However, the underlying mechanisms of resistant hypertension remain unclear.^3^

Emerging evidence suggests a strong link between hypertension and gut dysbiosis, as observed in both rodent models of hypertension and human hypertensive patients.^4^^,^^5^ Notably, the microbiota from hypertensive patients has been shown to increase blood pressure in germ-free mice upon fecal inoculation,^5^ indicating that gut bacteria play a role in blood pressure regulation. However, the precise mechanisms underlying this relationship remain unknown. Consistent findings support the view that a dysfunctional gut-brain axis contributes to hypertension, as increased sympathetic activity in the gut is associated with gut pathology, dysbiosis, and inflammation, further exacerbating hypertension.^6^^,^^7^ Interestingly, fecal microbiota transplantation from normotensive Wistar Kyoto rats (WKY) to spontaneously hypertensive rats (SHR) lowered blood pressure, partly by reducing neuroinflammation and sympathetic drive,^7^ highlighting gut microbiota as a novel target for blood pressure control.

Recent studies have shown that drugs that reduce neuroinflammation, such as minocycline, spironolactone, and mycophenolate, can improve gut dysbiosis and lower blood pressure.^8-10^ Furthermore, studies in hypertensive rodent models have shown that first-line antihypertensive drugs, such as captopril, losartan, and amlodipine, can alter the gut microbiota, and these changes may contribute to the observed reduction in blood pressure.^11-13^ These microbiota changes are associated with reduced sympathetic activity in the gut, improved gut integrity, and decreased permeability.^11^^,^^12^ However, while HCTZ induces significant microbiota changes due to its antibacterial activity,^14^these alterations do not contribute to its antihypertensive effect. Importantly, HCTZ failed to attenuate neuroinflammation and sympathetic gut overactivity, nor did it restore gut histopathology or barrier integrity in SHRs, underscoring its limited efficacy in addressing gut–brain axis dysfunction in this hypertensive model.^11^

The gut microbiota can also influence drug bioavailability and efficacy, as bacterial enzymatic activity may metabolize drugs into inactive forms or activate prodrugs.^15^^,^^16^ Studies have shown that gut microbiota depletion via antibiotics suppresses amlodipine metabolism ^17^ and enhances the antihypertensive effect of ester-based ACE inhibitors, such as quinapril.^18^ These findings suggest that manipulating the gut microbiota could regulate the pharmacokinetics and effectiveness of antihypertensive drugs in resistant hypertension. However, no data are currently available on the potential effects of the gut microbiota on the bioavailability of thiazide diuretics.

Meta-analyses of clinical trials have demonstrated a significant reduction in blood pressure among patients treated with probiotics.^19^ However, current clinical practice guidelines for arterial hypertension management do not include recommendations for probiotic use.^20^^,^^21^ The antihypertensive effect of probiotics, such as *Limosilactobacillus fermentum* CECT5716 (LC40), has been linked to restoring gut dysbiosis, improving gut integrity, and rebalancing T helper (Th)-17/regulatory T (Treg) cells, ultimately preventing endothelial dysfunction in various hypertensive rat models.^22^^,^^23^ Additionally, *Limosilactobacillus plantarum* has been shown to increase plasma levels of unmetabolized amlodipine, possibly due to enhanced absorption, although the underlying mechanism was not experimentally addressed in the original study.^24^ Based on these findings, we hypothesize that probiotic LC40 may enhance the antihypertensive effect of HCTZ by restoring gut dysbiosis and improving gut integrity. Accordingly, we evaluated whether LC40 treatment modulates the antihypertensive efficacy of HCTZ in SHRs, a genetic model of hypertension, with a focus on gut dysbiosis, the immune system, and the gut‒brain axis. Although HCTZ is not a prodrug susceptible to microbial inactivation, potential gut‒drug interactions could modulate its efficacy via unknown mechanisms. Therefore, we also measured plasma HCTZ levels to assess possible pharmacokinetic contributions to the observed effects.

## Results

### LC40 administration enhanced the antihypertensive and cardiac antihypertrophic effects of HCTZ without altering its plasma hydroelectrolytic adverse effect profile in SHR

**Figure 1.**
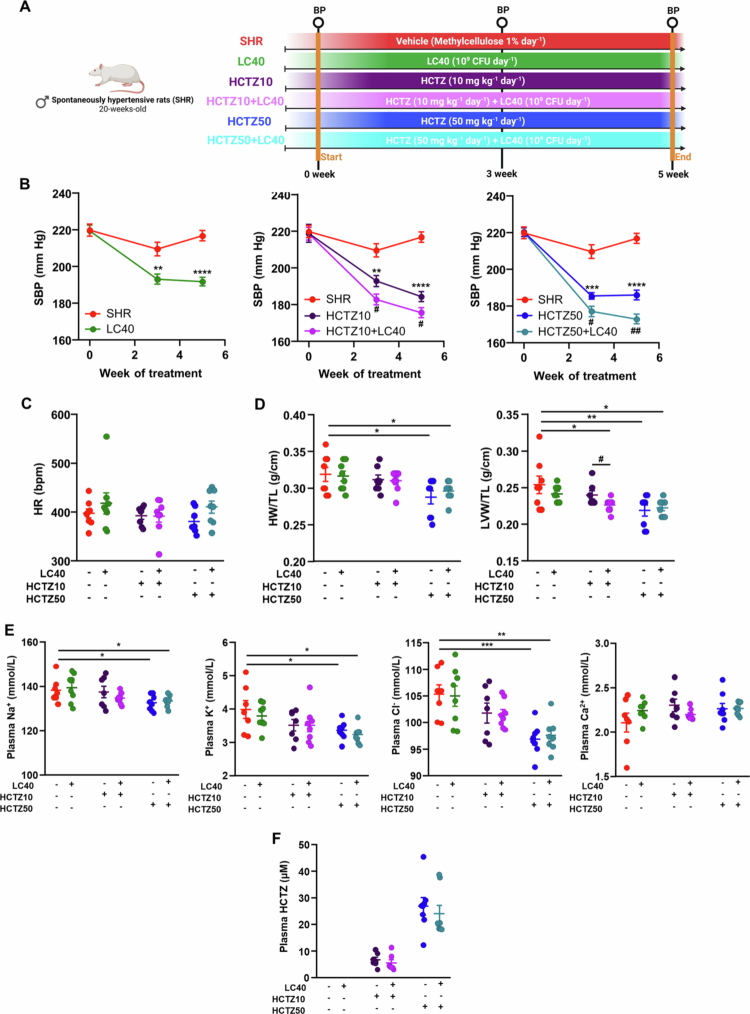
*Limosilactobacillus fermentum* CECT5716 (LC40) administration increased the antihypertensive and cardiac antihypertrophic effects of hydrochlorothiazide (HCTZ) without altering its pharmacokinetics and natriuretic effects. (A) Schematic illustration of the experimental protocol. Male spontaneously hypertensive rats (SHR) were divided into 6 groups: the untreated group (SHR, vehicle (methylcellulose 1%), 1 mL d^−1^), the treated group with LC40 (LC40, 10^9^ CFU d^−1^), the treated group with 10 mg kg^−1^ d^−1^ HCTZ (HCTZ10), the treated group with 50 mg kg^−1^ d^−1^ HCTZ (HCTZ50), the cotreated group with LC40 and HCTZ (HCTZ10 + LC40) and the cotreated group with LC40 and HCTZ (HCTZ50 + LC40) (BP: blood pressure measurement). Time course of systolic blood pressure (SBP) (B) and heart rate (HR) at the end of the study (C) measured by tail-cuff plethysmography. (D) Ratio of heart weight/tibia length (HW/TL) and left ventricle weight/tibia length (LVW/TL). (E) Plasma Na⁺, K⁺, Cl⁻ and Ca^2+^ concentrations. (F) HCTZ levels in plasma measured by ultra-performance liquid chromatography (UPLC). The values are expressed as the means ± SEM (*n* = 7−8). Tail cuff SBP determinations were analyzed by two-way repeated-measures ANOVA with Sidak's multiple comparison test. Other parameters were analyzed by one-way ANOVA with a Tukey's post hoc test. *p < 0.05, **p < 0.01, ***p < 0.001, and ****p < 0.0001 significant differences compared with the SHR group. ^#^*p* < 0.05 and ^##^*p* < 0.01 significant differences compared with the HCTZ10 or HCTZ50 group.

SHR were treated with LC40 alone or in combination with HCTZ at 10 or 50 mg/kg/d for 5 weeks (Figure 1A). As expected, LC40 administration led to a progressive decrease in systolic blood pressure (SBP) of ~25 mmHg. Similarly, HCTZ treatment at both doses reduced the SBP by ~30–34 mmHg. Notably, compared with HCTZ alone, the coadministration of the LC40 with HCTZ further amplified the SBP reduction by an additional ~8−13 mmHg (Figure 1B). No significant changes in heart rate (HR) were observed across the experimental groups (Figure 1C). Hypertension has been linked to the time-dependent development of hypertrophy in target organs such as the heart and kidneys. We found that the heart weight/tibia length (HW/TL) index was lower in the group receiving the higher dose of HCTZ compared to untreated animals (Figure 1D). Additionally, the left ventricle weight/tibia length (LVW/TL) ratio, a more sensitive marker of cardiac hypertrophy associated with hypertension,^25^ was reduced in groups that experienced more sustained and pronounced SBP reductions (HCTZ10 + LC40, HCTZ50, and HCTZ50 + LC40). Interestingly, while HCTZ10 alone failed to reduce left ventricular hypertrophy, its combination with the LC40 significantly attenuated this effect (Figure 1D). In contrast, no significant changes were observed in the right kidney weight/tibia length ratio, an index of renal hypertrophy (data not shown).

Thiazides exert their diuretic effect by inhibiting the Na⁺/Cl⁻ cotransporter in the renal distal convoluted tubule, leading to plasma electrolyte imbalances, primarily hyponatremia, hypochloremia, and hypokalemia.^26^ We observed a significant reduction in plasma Na⁺, K⁺, and Cl⁻ concentrations in HCTZ50-treated rats, which remained unchanged with LC40 coadministration (Figure 1E). Interestingly, LC40 administration did not affect the plasma HCTZ concentration (Figure 1F). Overall, these findings suggest that LC40 neither altered the pharmacokinetics nor the natriuretic effects of HCTZ but enhanced its antihypertensive effect, likely through extrarenal mechanisms.

### LC40 enhances HCTZ-mediated endothelial protection through redox and immune modulation in SHR

Chronic HCTZ treatment in hypertensive individuals has been associated with reduced total peripheral resistance, which is mediated by both indirect (sodium balance-related) and direct vasodilatory effects.^26^ Consistent with this, we found that HCTZ treatment significantly reduced the contractile response to phenylephrine (Phe). This inhibitory effect on vascular contractility remained unchanged with LC40 coadministration, suggesting no interference with the vasodilatory mechanisms triggered by the diuretic (Figure S1).

**Figure 2.**
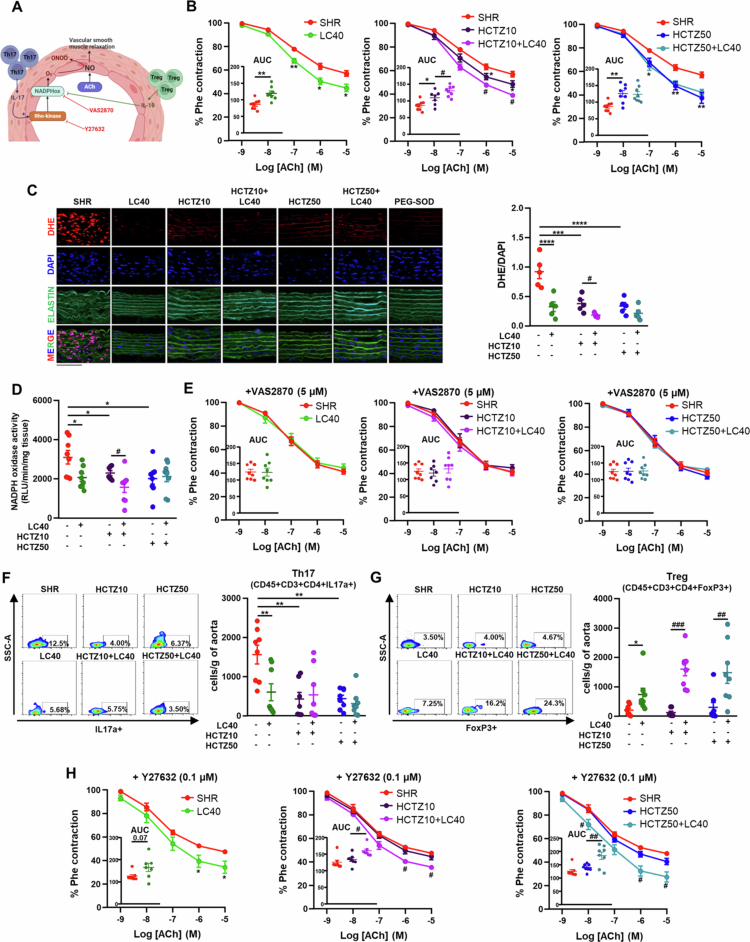
*Limosilactobacillus fermentum* CECT5716 (LC40) coadministration with hydrochlorothiazide (HCTZ) further improved endothelial dysfunction and aortic regulatory T cells (Treg) infiltration in spontaneously hypertensive rats (SHRs). (A) Schematic illustration of the mechanisms involved in endothelial dysfunction in SHR mediated by T helper (Th) cells. (B) Endothelium-dependent relaxation induced by acetylcholine (ACh) in aortas precontracted by phenylephrine (Phe). The inset graph displays the area under the relaxation curve (AUC). (C) Left images show arteries incubated in the presence of dihydroethidium (DHE), which produces red fluorescence when oxidized to ethidium by ROS, blue fluorescence intensity to nuclear staining with DAPI, and merged images including the green elastic layers. Moreover, in SHRs, slices were incubated for 30 min in the absence or presence of superoxide dismutase conjugated to polyethylene glycol (PEG-SOD, 25 U/mL). The graph on the right represents DHE staining normalized to DAPI. Bar scale: Scale bar: 50 μm. (D) NADPH oxidase activity measured by chemiluminescence to lucigenin. (E) Endothelium-dependent relaxation induced by ACh in aortas precontracted as described previously in the presence of the NADPH oxidase inhibitor VAS2870. The inset graph displays the AUC. (F) Infiltration of Th17 cells and (G) infiltration of Treg cells measured by flow cytometry in aortas from all experimental groups. (H) Endothelium-dependent relaxation induced by ACh in aortas precontracted as described previously, in presence of the Rho-kinase inhibitor Y27632. Groups: untreated group (SHR, vehicle (methylcellulose 1%) 1 mL d^−1^), treated group with 10^9^ CFU d^−1^ LC40 (LC40), treated group with 10 mg kg^−1^ d^−1^ HCTZ (HCTZ10), treated group with 50 mg kg^−1^ d^−1^ HCTZ (HCTZ50), co-treated group with LC40 and HCTZ (HCTZ10 + LC40) and co-treated group with LC40 and HCTZ (HCTZ50 + LC40). The values are expressed as the means ± SEM (*n* = 7−8). Aortic relaxations were analyzed by two-way repeated-measures ANOVA with Sidak's multiple comparison test. Other parameters were analyzed via one-way ANOVA with Tukey's post hoc test. *p < 0.05, **p < 0.01, ***p < 0.001, and ****p < 0.0001 significant differences compared with the SHR group. #*p* < 0.05, ^##^*p* < 0.01 and ^###^*p* < 0.001 significant differences compared with the HCTZ10 or HCTZ50 groups.

The antihypertensive properties of probiotics have been linked to improvements in endothelial dysfunction.^23^^,^^27^ To investigate whether this contributes to the enhanced antihypertensive effects of HCTZ induced by LC40, we examined the nitric oxide (NO)-dependent relaxation response to acetylcholine (ACh) in the aorta (Figure 2A). Aortae from SHR exhibited impaired endothelium-dependent vasodilation in response to ACh, which is indicative of endothelial dysfunction.^11^ This response was improved by LC40 and both doses of HCTZ (Figure 2B). Notably, in the HCTZ10 group, the relaxant response to ACh was further enhanced when LC40 was coadministered. The vasodilatory effects in all samples were completely abolished by L-NAME co-incubation, confirming the involvement of NO in the observed vasorelaxation (data not shown).

Aortic endothelial dysfunction in SHR is primarily attributed to reduced NO bioavailability due to reactive oxygen species (ROS) interference.^28^ To assess vascular ROS levels, we measured red fluorescence in aortic sections incubated with dihydroethidium (DHE). PEG-SOD abolished the red fluorescence in the SHR samples, confirming O₂⁻ specificity. Both the LC40 and HCTZ treatments reduced DHE staining, with LC40 coadministration further decreasing ROS levels in the HCTZ10 group (Figure 2C). Among the ROS-generating enzymes, NADPH oxidases play a key role in hypertension development. Interestingly, groups with lower ROS content also exhibited reduced NADPH oxidase activity, indicating that both LC40 and HCTZ suppress NADPH-driven ROS production (Figure 2D). Moreover, these inhibitory effects appear to contribute to improved endothelial function, as treatment with the selective NADPH oxidase inhibitor VAS2870 enhanced ACh-induced relaxation to a level comparable to that observed in the LC40 + HCTZ-treated group (Figure 2E).

Vascular T-cell infiltration has been implicated in ROS production and endothelial dysfunction in SHRs.^23^^,^^29^ Specifically, IL-17, which is secreted by Th17 cells, activates NADPH oxidase,^30^ while IL-10, which is released by Tregs, inhibits its activity (Figure 2A).^31^ We observed reduced aortic Th17 infiltration in all treated groups, though LC40 coadministration did not further decrease Th17 levels in HCTZ-treated rats (Figure 2F). In contrast, LC40 increased Treg infiltration, both when administered alone and in combination with HCTZ (Figure 2G). IL-17 has been linked to RhoA/Rho kinase-mediated endothelial dysfunction in SHRs.^11^ ACh-induced relaxation was improved in aortic rings from untreated SHR following incubation with the Rho kinase inhibitor Y27632 (Figure 2H). However, groups with higher Treg levels exhibited additional improvements in endothelium-dependent relaxation beyond those derived from Rho kinase inhibition alone. Overall, our findings demonstrate that LC40 coadministration further improves endothelial dysfunction by reducing NADPH oxidase-driven ROS production, likely through increased Treg infiltration.

### LC40 coadministration reshapes gut microbiota alterations induced by HCTZ in SHR

A well-established link exists between gut dysbiosis and elevated blood pressure in SHRs.^4^^,^^7^ Previously, we demonstrated that a high dose of HCTZ (90 mg kg^−1^ d^−1^) altered the gut microbiota composition at the phylum level, reducing Firmicutes while increasing Bacteroidetes, resulting in a lower Firmicutes/Bacteroidota (F/B) ratio in SHRs. Additionally, HCTZ treatment decreased the proportion of butyrate-producing bacteria. Indeed, the antihypertensive effects of HCTZ appeared to be independent of these microbiota changes, as fecal microbiota transplantation (FMT) from HCTZ-treated rats to untreated SHR failed to reduce blood pressure.^11^ Given that LC40 has been shown to induce beneficial modifications in gut dysbiosis under hypertensive conditions,^22^ we investigated whether it could alter HCTZ-induced microbiota changes.

**Figure 3.**
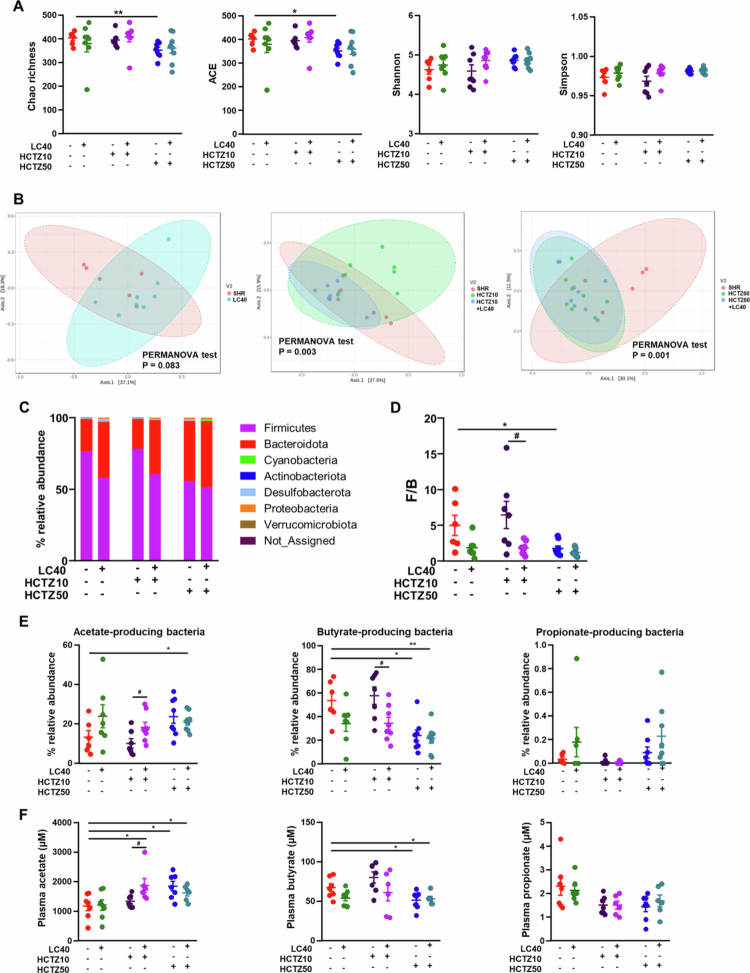
*Limosilactobacillus fermentum* CECT5716 (LC40), hydrochlorothiazide (HCTZ) and LC40 coadministration with HCTZ reshaped the remodeling of the gut microbiota in spontaneously hypertensive rats (SHRs). (A) The microbial DNA from fecal samples was analyzed by 16S rRNA gene sequencing. Ecological parameters of richness, such as Chao, and abundance-based coverage estimator (ACE), and diversity, such as the Shannon and Simpson. (B) Principal coordinate analysis (PCoA) of the gut microbiota from all experimental groups. (C) Phylum breakdown of the seven most abundant bacterial communities in the fecal samples was obtained from all experimental groups. (D) The Firmicutes/Bacteroidetes (F/B) ratio was calculated as a biomarker of gut dysbiosis. (E) Relative proportions of total acetate-, total butyrate- and total propionate-producing bacteria expressed as the relative abundance of total bacteria. (F) Acetate and butyrate plasma levels determined by gas chromatography. The values are expressed as the means ± SEM (*n* = 6−8). Groups: untreated group spontaneously hypertensive rats (SHR, vehicle (methylcellulose 1%) 1 mL d^−1^); treated group with 10^9^ CFU d^−1^ LC40 (LC40); treated group with 10 mg kg^−1^ d^−1^ HCTZ (HCTZ10); treated group with 50 mg kg^−1^ d^−1^ HCTZ (HCTZ50), co-treated group with LC40 and HCTZ (HCTZ10 + LC40); and co-treated group with LC40 and HCTZ (HCTZ50 + LC40). The data were analyzed via one-way ANOVA with Tukey's post hoc test. *p < 0.05 and **p < 0.01 significant differences compared with the SHR group. ^#^*p* < 0.05 significant differences compared with the HCTZ10 group.

The rarefaction curves for each sample demonstrated that the sequencing depth was sufficient to capture most of the microbial diversity in the samples, as all curves reached a clear plateau (Figure S2). To assess alpha diversity, we analyzed the Shannon and Simpson indices (ecological diversity parameters) along with the Chao and abundance-based coverage estimator (ACE) indices (richness parameters). No significant changes in alpha diversity were observed following the LC40 or HCTZ10 treatments. However, SHR treated with HCTZ50 presented reduced microbiota richness, which remained unaltered by LC40 coadministration (Figure 3A). Axonometric two-dimensional principal component analysis (PCoA) revealed that HCTZ treatment significantly reshaped the gut microbiota composition. Interestingly, the coadministration of LC40 with HCTZ10 modified the gut microbiota composition but did not prevent the changes induced by HCTZ50 (Figure 3B). At the phylum level, neither the LC40 nor the HCTZ10 induced significant shifts in phyla proportions. However, LC40 administration with HCTZ10 led to a reduction in Firmicutes and an increase in both Bacteroidota and Proteobacteria. Similar changes were observed in the HCTZ50 group, but LC40 coadministration did not significantly alter these effects (Table S1, Figure 3C). The F/B ratio, commonly used as a gut dysbiosis marker,^4^ was significantly reduced in the HCTZ10 + LC40, HCTZ50, and HCTZ50 + LC40 groups (Figure 3D).

Changes in populations of short-chain fatty acid (SCFA)-producing bacteria are another key indicator of gut microbiota dysbiosis in SHRs.^4^^,^^7^^,^^11^^,^^12^^,^^23^ We observed a reduction in butyrate-producing bacteria following HCTZ50 treatment, primarily affecting the *Lachnospiraceae_NK4A136_group* (*Lachnospiraceae*) (Figures S3, S4), with no changes in acetate- or propionate-producing bacteria (Figure 3E). Interestingly, LC40 coadministration with HCTZ10 increased the abundance of acetate-producing bacteria, primarily *Bacteroides* (*Bacteroidaceae*) and *Ruminococcus* (*Ruminocaccaceae*) (Figures S3, S4), but reduced the abundance of butyrate-producing bacteria (Figure 3E), mainly by decreasing the abundance of *Lachnospiraceae_NK4A136_group* bacteria (*Lachnospiraceae*) (Figures S3, S4). Moreover, the administration of LC40, either alone or in combination with HCTZ50, showed a tendency toward an increased relative abundance of propionate-producing bacteria (Figure 3E), predominantly *Akkermansia* (Figure S4)), in line with previous observations in obese murine models,^32^ although this effect did not reach statistical significance. These SCFAs are absorbed from the gut into the hepatic portal circulation and/or the lacteal lymphatic system before reaching the liver. Butyrate and propionate, which are metabolized primarily by hepatocytes, appear at low concentrations in the systemic circulation, while they exert effects on target organs.^33^ We observed increased plasma acetate concentrations in the HCTZ10 + LC40 group compared to the HCTZ10 group. Additionally, compared with no treatment, HCTZ50 increased acetate levels while reducing butyrate plasma concentrations, with no changes observed upon coadministration with LC40. No significant differences were detected in propionate plasma concentrations across all experimental groups (Figure 3F).

Using the Kyoto Encyclopedia of Genes and Genomes (KEGG) database, we assessed gut microbial functions across the study groups. KEGG modules associated with carbon metabolism, amino acid metabolism, lipid/isoprenoid biosynthesis, energy metabolism and LPS biosynthesis were altered by HCTZ treatment (Figure S5A). Interestingly, the HCTZ10 group presented an increase in the expression of genes related to LPS biosynthesis, while genes involved in the lipopolysaccharide (LPS) export system remained unchanged. Notably, these alterations were normalized with LC40 coadministration (Figure S5B).

### LC40 treatment improved gut pathology and permeability, and MLNs T cell profiles in SHR treated with HCTZ

**Figure 4.**
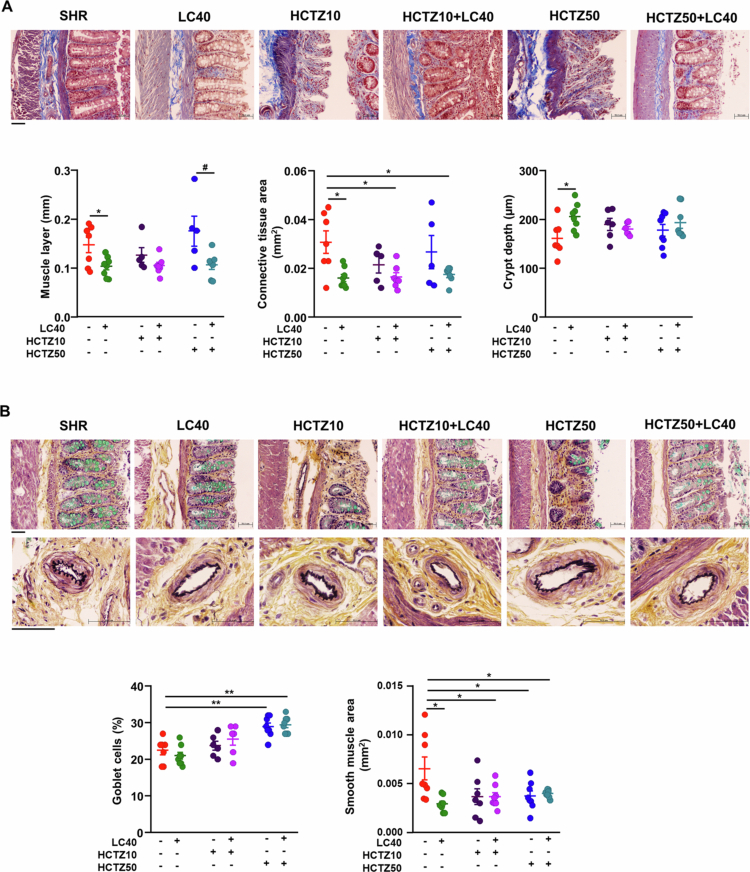
Effects of *Limosilactobacillus fermentum* CECT5716 (LC40), hydrochlorothiazide (HCTZ) and LC40 coadministration with HCTZ on the gut pathological alterations in the colon of spontaneously hypertensive rats (SHRs). (A) Representative micrographs of Masson-trichrome staining and quantitative analysis of muscle layer length, connective tissue area and crypt depth in the colon from all experimental groups. Scale bar: 50 μm. (B) Representative micrographs of Movat's pentachromic (MP) staining (upper) and representative micrographs of MP staining of the submucosal vascular smooth muscle layer (bottom) and quantitative analysis of the number of goblet cells per 100 epithelial cells and the area of the submucosal vascular smooth muscle layer in vessels less than 70 μm in the colon from all experimental groups. Scale bar: 50 μm. Note how focal damage to the glandular epithelium of the colon in the HCTZ10 and HCTZ50 groups was prevented with LC40 coadministration. The values are expressed as the means ± SEM (*n* = 5−8). The groups used were as follows: the untreated group, SHR (vehicle (methylcellulose 1%) 1 mL d^−1^); the treated group, 10^9^ CFU d^−1^ LC40 (LC40), (LC40); the treated group with 10 mg kg^−1^ d^−1^ HCTZ (HCTZ10), treated group with 50 mg kg^−1^ d^−1^ HCTZ (HCTZ50); the cotreated group, LC40 and HCTZ (HCTZ10 + LC40); and co-treated group with LC40 and HCTZ (HCTZ50 + LC40). The data were analyzed via one-way ANOVA with Tukey's post hoc test. *p < 0.05 and **p < 0.01 significant differences compared with the SHR group. ^#^*p* < 0.05 significant differences compared with the HCTZ50 group.

Increased gut pathology and epithelial permeability have been linked to the development of hypertension.^34-36^ LC40 treatment alleviated colonic thickening of the muscular layer, reduced the expansion of connective tissue, and increased the lower crypt depth observed in SHR (Figure 4A). Additionally, when coadministered with HCTZ, particularly HCTZ50, LC40 also mitigated muscular layer thickening and connective tissue expansion and reduced epithelial injury. Notably, both HCTZ50-treated groups exhibited an increased percentage of mucus-producing goblet cells (Figure 4B). Notably, the higher cross-sectional area of the colonic arterioles detected in SHR was reduced in all the treatment groups, indicating vascular antiproliferative effects, which were not observed with HCTZ10 alone (Figure 4B).

**Figure 5.**
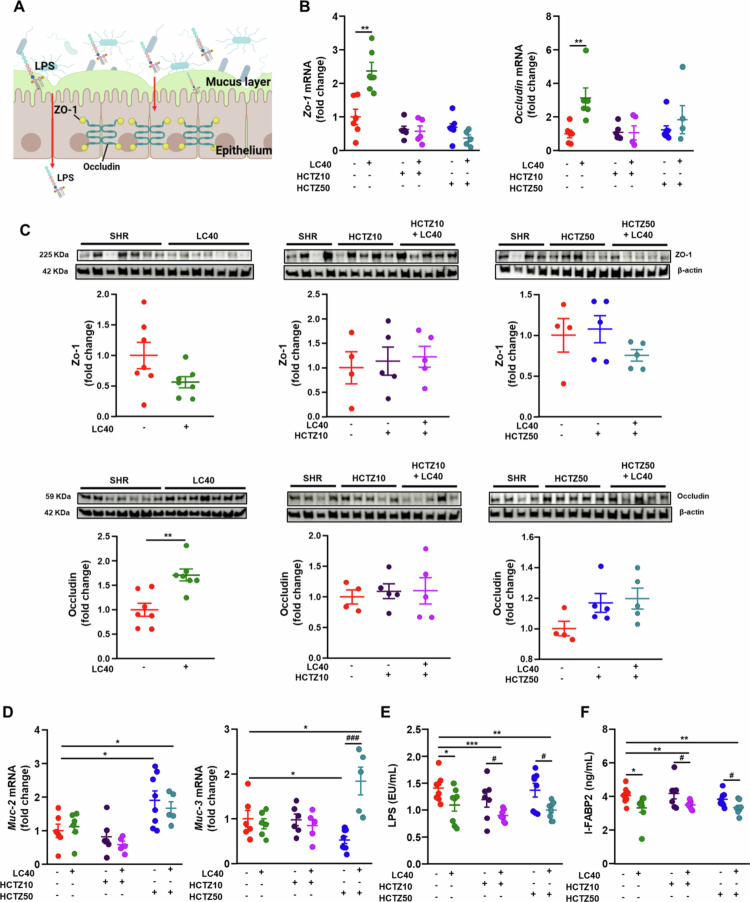
Effects of *Limosilactobacillus fermentum* CECT5716 (LC40), hydrochlorothiazide (HCTZ) and LC40 coadministration with HCTZ on gut integrity and permeability in spontaneously hypertensive rats (SHRs). (A) Schematic illustration of the role of tight junction proteins and the mucus layer in maintaining intestinal integrity, regulating permeability, and controlling the passage of bacterial metabolites, such as lipopolysaccharides (LPSs). (B) mRNA and (C) protein levels of occludin and zonula occludens-1 (ZO-1) in the colons of all the experimental groups. (D) mRNA levels of mucin *(Muc)-2* and *Muc-3* in the colons of all the experimental groups. (E) Plasma levels of endotoxin expressed as endotoxin units/mL (EU/mL). (F) Measurement of intestinal fatty acid-binding protein 2 (I-FABP2) in plasma. The values are expressed as the means ± SEM (*n* = 4−8). Groups: untreated group SHR (vehicle (methylcellulose 1%) 1 mL d^−1^), treated group with 10^9^ CFU d^−1^ LC40 (LC40), treated group with 10 mg kg^−1^ d^−1^ HCTZ (HCTZ10), treated group with 50 mg kg^−1^ d^−1^ HCTZ (HCTZ50), cotreated with LC40 and HCTZ (HCTZ10 + LC40) and cotreated with LC40 and HCTZ (HCTZ50 + LC40). Unpaired t-test was performed between SHR and LC40 groups. The data were analyzed via one-way ANOVA with Tukey's post hoc test. *p < 0.05, **p < 0.01 and ***p < 0.001 significant differences compared with the SHR group. ^#^*p* < 0.05, ^###^*p* < 0.001 significant differences compared with HCTZ groups.

Studies in hypertensive animal models suggest that intestinal barrier disruption occurs in SHR compared to WKY rats, but only after hypertension is established.^11^^,^^36^ When this critical barrier is compromised, microbial-derived substances such as LPS can translocate from the gut into the systemic circulation, where they bind to TLR4 and activate inflammatory pathways. To assess gut barrier integrity, we analyzed colonic mRNA levels and protein expression of key barrier-forming junction proteins, including occludin, zonula occludens-1 (*Zo-1*), and mucins *Muc-2* and *Muc-3* (Figure 5A). LC40 treatment increased *Zo-1* and *occludin* mRNA levels (Figure 5B); however, only occludin protein expression showed a detectable increase (Figure 5C). No other treatment group significantly altered the mRNA or protein expression of these junction proteins, suggesting that there were no major changes in barrier function. Additionally, high gut permeability in fully hypertensive SHR has been associated with reduced goblet cell numbers.^36^ Goblet cells secrete mucins, which protect the gut from pathogen infiltration and regulate the gut immune response as part of the physiological barrier.^37^ In line with the observed increase in goblet cell content, *Muc-2* mRNA levels—encoding the main secreted mucin—were elevated in both the HCTZ50 and HCTZ50 + LC40 groups. In contrast, *Muc-3* transcripts, which are expressed primarily in enterocytes and to a lesser extent in goblet cells, were reduced following HCTZ50 treatment but were partially restored with LC40 coadministration (Figure 5D). These findings suggest that *Muc-3* expression may reflect broader aspects of epithelial cell functionality beyond the goblet cell number. Although the modulation of *Muc-3* expression by LC40 could be indicative of improved epithelial status, these results alone do not support a conclusive protective effect on the mucus layer.

To further evaluate the integrity of the intestinal barrier, we measured plasma LPS concentrations and found that the LC40-treated groups exhibited reduced plasma LPS levels (Figure 5E). The lower LPS concentration in the HCTZ10 + LC40 group compared to the HCTZ10 group may be partially attributed to decreased LPS biosynthesis. Intestinal fatty acid-binding protein (I-FABP)2 is a well-established marker of gut permeability,^38^ and elevated circulating I-FABP2 levels have been observed in both animal models and humans with hypertension.^11^^,^^38^ Notably, groups with reduced plasma LPS concentrations also exhibited lower plasma I-FABP levels (Figure 5F).

Both LPS and SCFAs are known to influence immune cell populations associated with hypertension.^39^ Additionally, HCTZ treatment, which induces natriuresis, can prevent sodium-mediated differentiation and activation of Th17 cells.^40^ In our study, HCTZ-treated rats exhibited a reduced Th17 population in MLNs, while Treg and Th1 populations remained unchanged by HCTZ (Figure S6). Similarly, LC40 treatment alone decreased Th17 cells but did not alter the changes induced by HCTZ. However, all LC40-treated groups displayed an increased proportion of Tregs (Figure S6), with comparable changes observed in the spleen across all experimental groups (Figure S6).

### LC40 treatment mitigated neuroinflammation and sympathetic overactivity in the SHR

**Figure 6.**
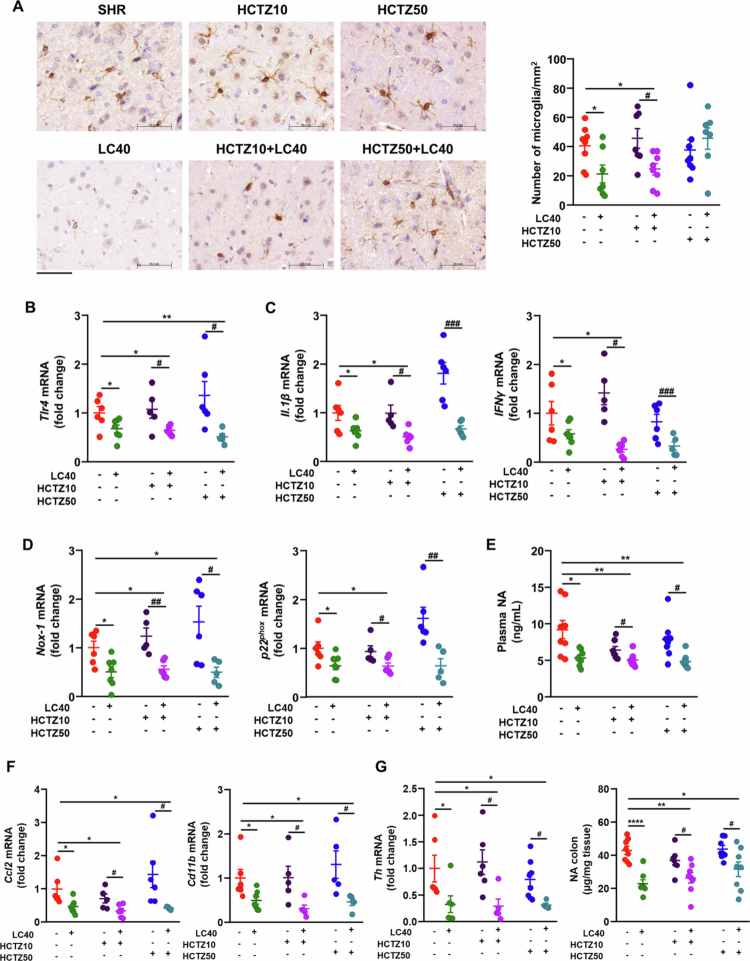
Effects of *Limosilactobacillus fermentum* CECT5716 (LC40), hydrochlorothiazide (HCTZ) and LC40 co-administration with HCTZ on microglia, neuroinflammation in the paraventricular nucleus (PVN) of the hypothalamus in spontaneously hypertensive rats (SHR). (A) The upper images show the immunohistochemical expression of the iba1 marker, which can be used to identify microglia in different treatment groups. Scale bar: 50 μm. (B) mRNA levels of toll-like receptor (*Tlr*)-4 and (C) proinflammatory cytokines, interleukin (*Il*)-1β and interferon (*Ifn*)-*γ* in homogenates from the brain PVN. (D) mRNA levels of the NADPH oxidase subunits *Nox-1* and *p22*^*phox*^ in the PVN. (E) Plasma noradrenaline (NA) content. (F) mRNA levels of monocyte chemotactic protein (*Ccl2*) and *Cd11b* in the PVN. (G) Colonic tyrosine hydroxylase (*Th*) expression and the colonic NA concentration in all experimental groups. The values are expressed as the means ± SEM (*n* = 5−8). Groups: untreated group SHR (vehicle (methylcellulose 1%) 1 mL d^−1^), treated group with 10^9^ CFU d^−1^ LC40 (LC40), treated group with 10 mg kg^−1^ d^−1^ HCTZ (HCTZ10), treated group with 50 mg Kg^−1^ d^−1^ HCTZ (HCTZ50), co-treated group with LC40 and HCTZ (HCTZ10 + LC40) and co-treated group with LC40 and HCTZ (HCTZ50 + LC40). The data were analyzed via one-way ANOVA with Tukey's post hoc test. *p < 0.05, **p < 0.01 and ****p < 0.0001 significant differences compared with the SHR group. ^#^*p* < 0.05, ^##^*p* < 0.01 and ^###^*p* < 0.001 significant differences compared with the HCTZ groups.

Hypertension has been linked to increased microglial activation, oxidative stress, and neuroinflammation in autonomic brain regions.^41^ In the paraventricular nucleus (PVN) of the hypothalamus, a key autonomic brain region involved in blood pressure regulation, the total number of microglial cells was higher in SHRs than in WKY rats.^11^^,^^42^ LC40 treatment reduced microglial cell counts in SHRs, whereas both doses of HCTZ failed to alter microglial cell content; however, coadministration of HCTZ10 with LC40 led to a significant reduction in these cells in the PVN (Figure 6A). Microglial activation by agents such as LPS induces a proinflammatory phenotype. Toll-like receptor (TLR)4 activation in the brainstem contributes to neuroinflammation and increased sympathetic outflow.^43^^,^^44^ Consistent with lower plasma LPS levels, our results revealed that *Tlr4* expression in the PVN was reduced in all groups treated with LC40 (Figure 6B), which was associated with decreased mRNA levels of proinflammatory cytokines such as interleukin *(Il)-1β* and interferon *(Ifn)γ* (Figure 6C).

Excessive oxidative stress in the PVN, driven by NADPH oxidase activity, is a key factor in the elevation of central sympathetic outflow.^45^ In SHRs, we observed reduced mRNA levels of the NADPH oxidase subunits *Nox1* and *p22*^*phox*^ in groups treated with LC40 compared to those receiving HCTZ alone (Figure 6D). To assess the effects of these treatments on sympathetic outflow, we measured plasma noradrenaline (NA) levels. While HCTZ treatment alone did not affect plasma NA concentrations, all SHR groups treated with LC40 exhibited a significant reduction in NA levels (Figure 6E), indicating decreased sympathetic activity.

Elevated sympathetic activity also impacts the bone marrow (BM), leading to an increased number of inflammatory cells that migrate to the PVN and exacerbate neuroinflammation.^46^^,^^47^ Consistently, we observed heightened inflammation in the PVN of SHR and SHR treated with HCTZ, associated with increased mRNA levels of *Ccl2*, which facilitates BM cell infiltration into the brain parenchyma, and *Cd11b*, a general marker of myeloid-lineage cells, including macrophages, neutrophils, and activated microglia, that contribute to neuroinflammation. Importantly, the expression of these markers was reduced in all SHR groups treated with LC40 (Figure 6F).

Furthermore, excessive sympathetic activity affects gut physiology in SHRs.^6^ We found that the mRNA levels of tyrosine hydroxylase (*Th*), an enzyme essential for NA synthesis, as well as NA concentrations in the colon (Figure 6G), remained unchanged with HCTZ treatment but were significantly reduced following coadministration with LC40. Overall, our findings suggest that LC40 enhances gut barrier function, thereby reducing endotoxemia, preventing neuroinflammation in the PVN, and ultimately lowering systemic sympathetic tone. This reduction in intestinal sympathetic activity is associated with improved permeability. Thus, bidirectional gut–brain communication appears to play a crucial role in the protective effects of LC40.

### Changes in gut microbiota composition played a key role in mediating the enhanced antihypertensive effect of HCTZ when co-administered with LC40

**Figure 7.**
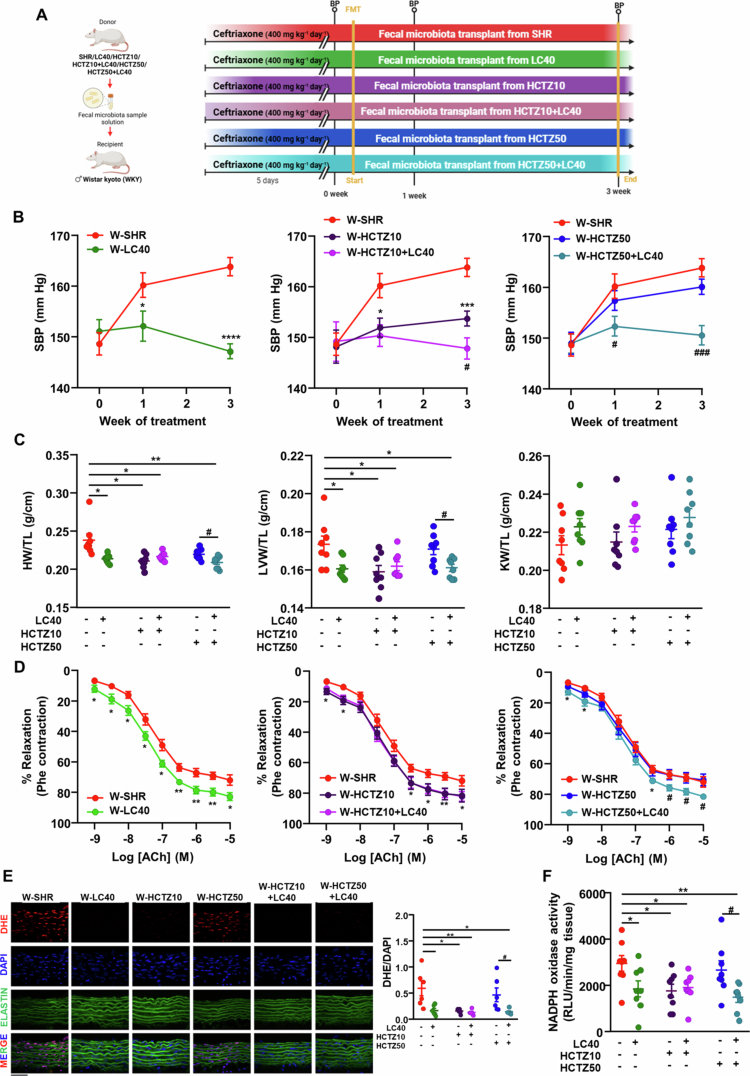
Effects of fecal microbiota transplantation (FMT) from treated spontaneously hypertensive rats (SHR) to normotensive Wistar Kyoto rats (WKYs). (A) Schematic illustration of FMT and experimental design. Fecal sample solutions from untreated and treated donor SHR were administered to recipient WKY rats for 3 consecutive days and once every 3 d for a total extension of 3 weeks after 5 consecutive days of ceftriaxone treatment to eliminate the microbiota. WKY rats were divided into 6 groups: FMT from SHR to WKY (W-SHR), from LC40 to WKY rats (W-LC40), from the HCTZ10 group to WKY rats (W- HCTZ10), from the HCTZ10 + LC40 group to WKY rats (W-HCTZ10 + LC40), from the HCTZ50 group to WKY rats (W- HCTZ50) and from the HCTZ50 + LC40 group to WKY rats (W-HCTZ50 + LC40). (B) Time course of systolic blood pressure (SBP) measured by tail-cuff plethysmography. (C) Ratio of heart weight/tibia length (HW/TL), left ventricle weight/tibia length (LVW/TL) and kidney weight/tibia length (KW/TL). (D) Endothelium-dependent relaxation induced by acetylcholine (ACh) in aortas precontracted by phenylephrine (Phe). (E) Pictures showing arteries incubated in the presence of dihydroethidium (DHE), which emits red fluorescence when oxidized to ethidium by ROS, blue fluorescence intensity to nuclear staining with DAPI, and merged images including the green elastic layers. Scale bar: 50 μm. (F) NADPH oxidase activity measured by chemiluminescence to lucigenin. Values are expressed as mean ± SEM (*n* = 6−8). Tail cuff SBP determinations and aortic relaxations were analyzed by two-way repeated-measures ANOVA with Sidak's multiple comparison test. Other parameters were analyzed via one-way ANOVA with Tukey's post hoc test. *p < 0.05, **p < 0.01, ***p < 0.001 and ****p < 0.0001 significant differences compared with the W-SHR group. ^#^*p* < 0.05 and ^###^*p* < 0.001 significant differences compared with W-HCTZ groups.

To investigate the involvement of the microbiota in this enhanced antihypertensive effect, we performed a fecal microbiota transplant (FMT) from the different experimental groups into normotensive WKY rats (Figure 7A). The composition of the gut microbiota following FMT was assessed. The rarefaction curves for all the samples reached clear plateaus, indicating that the sequencing depth was sufficient to capture the majority of the microbial diversity (Figure S7). FMT from both untreated and treated SHR donors to WKY recipients influenced alpha diversity indices, with reduced richness, as reflected by the Chao and ACE estimators, while the Shannon and Simpson indices remained stable (Figure S8A). Nevertheless, no statistically significant differences in alpha diversity parameters were detected across the experimental groups. Similarly, the beta diversity differences observed between the untreated and treated SHR groups were no longer evident three weeks after FMT in the WKY recipients (Figure S8B).

At the phylum level, a decrease in Actinobacteriota was observed in the W-HCTZ10 group compared with the W-SHR group, whereas an increased relative abundance of Verrucomicrobiota was detected in the W-HCTZ50 + LC40 group compared with the W-HCTZ50 group (Figure S8C). No significant changes in the F/B ratio were detected across the FMT groups (Figure S8D). Notably, acetate-producing bacteria were enriched in W-HCTZ50 + LC40 relative to W-HCTZ50, while SHR donor groups treated with LC40 consistently showed a higher abundance of propionate-producing bacteria (predominantly *Akkermansia,* Figure S9B) than did their untreated counterparts after FMT into WKY recipients (Figure S8E).

As expected, FMT from SHR to WKY led to a progressive increase in SBP, reaching approximately 15  mmHg after three weeks. The SBP increase induced by FMT from SHR treated with HCTZ10 was significantly lower than that caused by untreated SHRs, whereas the increase observed after FMT from SHR treated with HCTZ50 was comparable to that of untreated SHRs. Notably, FMT from SHR treated with LC40, either alone or in combination with HCTZ, did not alter SBP (Figure 7B). No significant changes in HR were observed across the experimental groups (data not shown). Lower HW/TL and LVW/TL indices were associated with lower final SBP values, while the KW/TL index remained unchanged. Interestingly, when donor SHR were cotreated with LC40, FMT-induced cardiac hypertrophy in recipient WKY rats was lower than that observed with FMT from SHR treated with HCTZ50 alone (Figure 7C).

The contraction response to Phe remained unchanged among all experimental groups (Figure S10), suggesting that the vascular inhibition induced by HCTZ in SHR was independent of the microbiota and likely due to a direct effect on the vascular wall. However, endothelium-dependent relaxation in response to ACh was greater in the aortae of WKY rats receiving microbiota from the SHR-LC40 group compared to those receiving microbiota from untreated SHRs. Additionally, FMT from the HCTZ10-treated group, but not from the HCTZ50-treated group, improved ACh-induced relaxation in WKY rats. Remarkably, FMT from rats receiving the HCTZ50-LC40 combination further improved ACh-induced relaxation compared to FMT from HCTZ50-treated SHR alone (Figure 7D). These findings suggest that the improvement in endothelial function induced by LC40 treatment is transferable via the microbiota.

Increased endothelium-dependent relaxation was associated with reduced ROS content (Figure 7E) and decreased NADPH oxidase activity in the aorta (Figure 7F). Notably, pharmacological inhibition of NADPH oxidase with VAS2870 also improved ACh-induced relaxation in the W-SHR and W-HCTZ50 groups, reaching levels comparable to those in the LC40-treated groups (Figure S11). Given the critical role of Tregs in endothelial function and the increased aortic Treg infiltration observed in SHR following LC40 treatment, we further examined the Treg content in MLNs, the spleen, and the aorta. Interestingly, FMT from LC40-treated SHRs, either alone or in combination with HCTZ, increased the Treg populations to these secondary lymphoid organs and the aorta (Figure S12A). Strikingly, incubation of aortic rings with an IL-10 neutralizing antibody targeting the key cytokine released by Tregs that inhibits NADPH oxidase abolished the improvement in endothelial dysfunction induced by microbiota from LC40-treated SHR in WKY rats (Figure S12B). This highlights the critical role of Treg infiltration in this protective effect.

Overall, the hypertensive phenotype was transmissible via the microbiota of SHRs, but this transmission was prevented when the donor rats received LC40 treatment. The protective effects of microbiota from LC40-treated rats on endothelial dysfunction appear to be mediated by its ability to polarize T-helper (Th) cells toward a Treg phenotype in secondary lymphoid organs, thereby promoting vascular Treg infiltration.

## Discussion

The presence or absence of certain bacteria can significantly impact the bioavailability and effectiveness of specific drugs, such as those used to treat hypertension.^11^^,^^48^ The thiazide diuretic HCTZ lowers blood pressure but fails to reduce neuroinflammation, sympathetic activity, and gut dysbiosis in SHRs. In contrast, other classes of antihypertensive drugs, such as calcium channel blockers, angiotensin II receptor antagonists, and angiotensin-converting enzyme inhibitors, have been shown to improve these hypertension-related conditions.^11^^,^^12^ Our findings provide the first evidence that LC40 coadministration enhances the antihypertensive and cardiac antihypertrophic effects of HCTZ without exacerbating its plasma hydroelectrolytic adverse effects in SHRs. The additional reduction in SBP observed with LC40 and HCTZ coadministration suggests a potential extrarenal mechanism contributing to the increased blood pressure-lowering effect. Since LC40 did not alter HCTZ plasma concentrations or its natriuretic effects, the improvement in the antihypertensive response was not mediated by increased systemic drug exposure. Instead, its beneficial actions may be mediated through alternative extrarenal mechanisms, including endothelial protection, gut microbiota modulation, and reduced sympathetic activity.

Hypertension-induced endothelial dysfunction, characterized by impaired NO-dependent vasodilation,^28^ was improved by both LC40 and HCTZ. Notably, LC40 enhanced HCTZ10-induced improvements in endothelial function, likely by reducing ROS levels and suppressing NADPH oxidase activity. The observed increase in Treg infiltration in the LC40-treated groups further supports the role of immune modulation in these vascular benefits. Indeed, IL-10, the primary cytokine released by Treg cells, inhibits NADPH oxidase and improves endothelial function.^49^ These findings align with previous reports linking probiotic interventions with reduced oxidative stress and improved endothelial function in hypertensive models.^22^^,^^27^^,^^50^ Furthermore, the direct effects of acetate in the aorta may contribute to the endothelial protective effects of HCTZ in combination with LC40, as previously described.^7^

Gut microbiota changes played a crucial role in mediating the enhanced antihypertensive effects of HCTZ when co-administered with LC40. When combined with HCTZ, LC40 improved key characteristics of gut dysbiosis in SHRs,^4^ including a reduction in the F/B ratio and an increase in acetate-producing bacteria, leading to higher plasma acetate levels. While HCTZ50 alone altered the gut microbiota composition by reducing the F/B ratio and decreasing the number of butyrate-producing bacteria, LC40 selectively modulated these changes by increasing the number of acetate-producing bacteria and expanding the Treg population. Interestingly, coadministration of LC40 with HCTZ increased the relative abundance of *Bacteroides,* which are known to expand Treg populations in the gut,^51^ and may partially explain the increased Treg content in secondary lymphoid organs. SCFAs are key metabolites for maintaining intestinal homeostasis, serving as an energy source for intestinal epithelial cells and strengthening gut barrier function.^52^ Our findings on gut integrity are consistent with previous data showing that LC40 alone enhances gut integrity and reduces permeability in SHRs.^23^ However, the ability of LC40 alone to improve gut integrity was somewhat limited when co-administered with HCTZ. Nevertheless, the normalization of LPS biosynthesis pathways and increased MUC-3 production further support a beneficial role in reducing gut permeability. The observed reductions in plasma LPS and I-FABP2 levels in the LC40-treated groups further indicate enhanced gut barrier integrity, which may have contributed to the observed reductions in systemic inflammation and improved vascular function.^53^ In our study, both the ACE and Chao1 indices of alpha diversity were significantly reduced following HCTZ administration, indicating a decrease in microbial richness. Interestingly, LC40 coadministration did not restore these indices, suggesting that its beneficial effects are not necessarily linked to increased global diversity. Rather, the impact of LC40 may rely on specific compositional shifts, such as the preservation or enrichment of taxa involved in gut barrier maintenance, anti-inflammatory activity, and host–microbiota signaling. FMT from SHR to WKY resulted in pronounced alterations in the engrafted microbial community relative to that of the donors, indicating that the transplanted microbiota undergoes ecological adaptation to the intestinal environment of normotensive hosts. This adaptive process was particularly evident in the LC40-pretreated groups, where an expansion of taxa such as *Akkermansia*, a mucin-degrading bacterium with antihypertensive properties,^54^ was detected. Given that mucin production is intrinsically higher in WKYs than in SHRs,^34^ this host-derived factor may provide a selective niche that facilitates the engraftment and expansion of mucin-utilizing bacteria. Notably, FMT from SHR treated with LC40 and HCTZ led to significant improvements in SBP and endothelial function in recipient WKY rats, reinforcing the role of gut-derived factors in blood pressure regulation. Plasma SCFA concentrations reflect the net balance between microbial production and host-related processes such as intestinal absorption, epithelial transport, hepatic metabolism, and systemic clearance. In this study, we prioritized the analysis of plasma SCFA levels given their greater relevance to systemic effects, particularly those related to the regulation of endothelial function.^55^ Notably, groups with higher plasma acetate levels exhibited improved endothelial function, whereas this effect appeared attenuated in groups with lower plasma butyrate levels.

Neuroinflammation and sympathetic overactivity are key contributors to hypertension pathophysiology.^46^^,^^47^ Interestingly, for the first time, we described that probiotics consumption, such as LC40, reduced these processes under hypertensive conditions. An imbalance in SCFA-producing bacteria and altered plasma LPS concentrations has been linked to increased neuroinflammation in hypertension.^7^ The central nervous system and the digestive system are closely interconnected, with the brain playing a crucial role in maintaining gut health and homeostasis.^56^ The observed reductions in PVN microglial activation, proinflammatory cytokine expression, and NADPH oxidase activity suggest that LC40 exerts neuroprotective effects. These changes were accompanied by decreased plasma and colonic NA levels, indicating reduced sympathetic drive. Given that gut dysbiosis has been associated with heightened sympathetic activity, the microbiota-modulating effects of LC40 may have contributed to its ability to lower systemic sympathetic tone.

In conclusion, our study highlights the multifaceted benefits of LC40 in potentiating the antihypertensive, endothelial-protective, and gut-modulating effects of HCTZ in SHRs. These effects are likely mediated by reductions in oxidative stress, immune modulation, alterations in the gut microbiota, and the suppression of sympathetic activity. To our knowledge, this study provides the first evidence that probiotics can increase the efficacy of antihypertensive drugs without increasing adverse effects, suggesting a potential therapeutic strategy for resistant hypertension. Further research is warranted to explore the long-term cardiovascular benefits of LC40 and its potential clinical application as an adjunct therapy for hypertension management. Although our findings provide mechanistic insights into the effects of LC40 in a preclinical model, further validation in patient cohorts will be essential to confirm these results and evaluate their clinical relevance. Notably, recent reports have shown that probiotics, when administered alongside conventional antihypertensive therapy, significantly reduce both systolic and diastolic blood pressure in children with primary hypertension while maintaining an acceptable safety profile.^57^

Although our FMT protocol allowed for the transfer of hypertensive or treatment-modified microbiota to normotensive WKY rats, several limitations must be considered. First, the fecal material was stored at -80 °C prior to transplantation without the use of cryoprotectants or confirmed anaerobic processing conditions, which may have compromised the viability of anaerobic commensals. Second, fecal material was pooled from individual donors within each group, which, while intended to reduce individual variability, may mask relevant interindividual microbiota differences. In addition, we did not perform quality control analyses (e.g., microbial viability or pathogen screening) of the inoculum prior to transplantation. These factors, considering recent GRAFT (Guidelines for Reporting on Animal Fecal Transplantation) recommendations,^58^ represent relevant methodological constraints that could influence engraftment efficiency and microbiota‒host interactions. Therefore, while our results suggest a microbiota-dependent component in blood pressure regulation, caution is required in the interpretation of causality. Another limitation of our study is that we were not able to identify specific bacterial taxa that were selectively removed after HCTZ, L40, or HCTZ + L40 intervention; instead, our results reflect global shifts in microbial composition.

## Material and methods

### Animals and experimental groups

Experimental procedures involving animals were carried out in line with Directive 2010/63/EU of the European Parliament on the protection of animals used for scientific purposes and adhered to the ARRIVE reporting guidelines.^59^ Ethical approval was granted by the Ethics Committee for Animal Welfare at the University of Granada (Spain; permit number 16/02/2022/013/A).

We selected SHR as an animal model of polygenetic hypertension since it is similar to essential hypertension in nonobese people. Male rats were used because their blood pressure levels are greater than those of age-matched female animals.^60^ Twenty-week-old male SHR and WKY rats were obtained from Janvier Labs (Le Genest-Saint-Isle, Saint Berthevin Cedex, France) for use in the study. The study was performed to create equal-sized groups with enough statistical power. The person conducting the research was blinded to the drug treatment until data processing, and animals were randomized to treatment groups.

*Experiment 1*: Twenty-weeks-old male SHR were randomly divided into 6 groups: a) untreated SHR (SHR, 1 mL of vehicle (methylcellulose 1%) d^−1^, *n* = 8), b) SHR treated with LC40 (LC40, 10^9^ CFU d^−1^ by oral gavage for five weeks, *n* = 8),^23^ c) SHR treated with HCTZ (HCTZ10, 10 mg Kg^−1^ d^−1^ by oral gavage for five weeks, *n* = 8), d) SHR treated with HCTZ (HCTZ50, 50 mg Kg^−1^ d^−1^ by oral gavage for five weeks, *n* = 8), e) SHR co-treated with LC40 and HCTZ (HCTZ10 + LC40, *n* = 8), f) SHR co-treated with LC40 and HCTZ (HCTZ50 + LC40, *n* = 8). The doses of HCTZ selected were inferior to those used previously ^11^ to investigate whether coadministration of probiotics could increase the antihypertensive effect of HCTZ. LC40 was obtained from Biosearch S.A. (Granada, Spain) and routinely cultured in de Man, Rogosa, and Sharpe (MRS) media at 37 °C under anaerobic conditions using the Anaerogen system (Oxoid, Basingstoke, Hants, UK). For probiotic administration, the lyophilized bacterial preparation (2 × 10¹¹ CFU g⁻ ¹ ) was aliquoted and stored at –20 °C until use. Prior to administration, the bacteria were resuspended in tap water and immediately delivered to the animals. The selected LC40 dose was based on previous studies conducted in SHRs.^23^

*Experiment 2*: To explore the involvement of the microbiota in BP regulation, FMT to normotensive twenty-week-old male WKY was performed.^7^ For this purpose, in the morning, the animals from all the groups in experiment 1 were restrained in clean plastic tubes, and fecal samples were collected directly in sterile Eppendorf tubes and immediately stored at –80 °C. Then, similar weights of fecal samples from all individual rats in each group were pooled and used to generate a bacterial suspension by vigorously vortexing 1:20 in sterile phosphate-buffered saline (PBS) and centrifuging at 60 × g for 5 min to eliminate detritus. The suspension was aliquoted and stored at −80 °C. All these procedures were performed under aerobic conditions. Starting 1 week before administration, the recipient rats were administered 1 mL of ceftriaxone sodium (400 mg k^g−1^ d^−1^) daily for five consecutive days by oral gavage. Forty-eight hours after the last antibiotic treatment, the recipient rats were orally gavaged with the donor fecal contents (1 mL) immediately after thawing. The donor fecal contents were administered for 3 consecutive days and once every 3 d for a total extension of 3 weeks. The animals were randomly assigned to seven different groups of 8 animals each: WKY with SHR microbiota (W-SHR), WKY with microbiota from the LC40 group (W-LC40), WKY with microbiota from the HCTZ10 group (W-HCTZ10), WKY with microbiota from the HCTZ50 group (W-HCTZ50), WKY with microbiota from the HCTZ10 + LC40 group (W-HCTZ10 + LC40), and WKY with the HCTZ50 + LC40 group microbiota (W- HCTZ50 + LC40). At the end of the experiment, FMT engraftment was confirmed through 16S rRNA analysis of fecal samples.

In both experiments, all the animals were housed under standard laboratory conditions with dust-free laboratory bedding and enrichment and were cohoused with a maximum of 4 per cage. The animals were provided with water and food ad libitum. Water was changed every day, and both water and food intake were recorded every day.

### Blood pressure measurements

Prior to the experimental procedure, the rats were habituated for two weeks to the handling procedures required for vehicle administration and noninvasive blood pressure measurements. SBP and HR were measured in the morning in nonanesthetized, restrained rats using tail-cuff plethysmography (Digital Pressure Meter, LE5001; Letica S.A., Barcelona, Spain).^11^ To facilitate blood flow, the animals were prewarmed at 35 °C for 10–15 min before measurement. During the procedure, the rats were placed in a restraining plastic tube, and the tail was inserted through a pneumatic rubber cuff, which was then inflated with air. SBP was defined as the pressure at which the first pulse reappeared after occlusion. A minimum of 10 consecutive measurements were recorded per session, and the average of these values was used as the final SBP for each animal.

### Tissue collection and cardiac and renal weight indices

Following the trial, the rats were fasted overnight before being given 2.5 mL kg^−1^ equitensin intraperitoneally (i.p.). One hour after the last drug administration, the animals were exsanguinated, and blood was collected from the abdominal aorta. Next, the colon, kidneys and heart were dissected and weighed. The cortex from the kidney was collected, and the heart was divided into the left and right ventricles. All the tissues were preserved by freezing them in liquid nitrogen and then storing them at −80°C.

### Vascular reactivity studies

Thoracic aortic rings (3 mm) were mounted in organ chambers with Krebs solution (in mM: NaCl 118, NaHCO_3_ 25, glucose 11, KCl 4.75, CaCl_2_ 2, KH_2_PO_4_ 1.2, MgSO_4_ 1.2) and infused with carbogen at 37 °C. The rings were subjected to a resting tension of 2 g, and isometric tension was recorded using an isometric force‒displacement transducer (Letigraph 2000) coupled to an acquisition system, as previously described.^10^ Concentration‒response curves for Phe (10^−9^–10^−5^ M) were generated. The relaxation curves to ACh (10^−9^‒10^‒5^ M) were evaluated in aorta rings precontracted with Phe (10  μM). Further curves were generated via incubation with or without a non-selective competitive inhibitor of NADPH oxidase (VAS2870, 5 μM) or a Rho kinase inhibitor (Y27632, 0.1 μM) during 30 min. Some aortic rings were incubated with either a saline solution or a neutralizing IL-10 antibody (10  μg/mL) for 3 h before constructing a concentration–response curve to ACh in rings precontracted with 3 μM Phe. The results are presented as a percentage of precontraction tension levels.

### Vascular reactive oxygen species (ROS) content

To quantify vascular ROS, the American Heart Association recommends fluorescence analysis with DHE as a useful approach to measure and image intracellular superoxide (O_2_^−^) production from tissues because of its high sensitivity, which should be complemented with a second, independent assay, such as the lucigenin chemiluminescence assay with appropriate controls.^61^

For in situ ROS detection by DHE fluorescence, unfixed thoracic aortic rings were cryopreserved (PBS, 0.1 mol/L, plus 30% sucrose for 1–2 h), included in optimum cutting temperature compound medium (Tissue-Tek; Sakura Finetechnical, Tokyo, Japan), frozen (−80 °C), and 5 μm cross sections were obtained in a cryostat (Microm International Model HM500 OM). The sections were incubated for 15 min in HEPES-buffered solution containing DHE (10^−6^ M), counterstained with the nuclear stain DAPI (3 × 10^−7^ M) and subsequently examined for 24  h via a fluorescence microscope (Leica DM IRB, Wetzlar, Germany). The sections were photographed, and fluorescence was quantified using ImageJ software. All the parameters (pinhole, contrast, gain, offset) were held constant for all the sections from the same experiment. All images presented in the manuscript were acquired using identical acquisition settings, including different laser intensities and exposure times for each fluorescence channel, to ensure consistency and comparability across conditions. ROS production was estimated from the ratio of ethidium/DAPI fluorescence.^62^ DHE fluorescence was also analyzed in aortic sections from the SHR group incubated for 30 min at 37 °C in the presence of superoxide dismutase conjugated to polyethylene glycol (PEG-SOD, 25 U/mL) before DHE.

We used the lucigenin-enhanced chemiluminescence assay to evaluate NADPH oxidase activity in intact aortic segments as previously described.^11^ Aortic rings from all experimental groups were placed in HEPES-containing physiological salt solution (pH 7.4): CaCl_2_ 1.2, glucose 5.5, HEPES 20, KCl 4.6, KH_2_PO_4_ 0.4, MgSO_4_ 1, NaCl 119, NaHCO_3_ 1 (in mM) for 30 min at 37 °C. Luminescence was measured after the addition of NADPH (100 μM) and lucigenin (5 μM), and measurements were taken over 200 s in 5-s intervals in a scintillation counter (Lumat LB 9507, Berthold, Germany). Enzyme activity was calculated by subtracting the basal values from the NADPH-treated values, and the results are expressed as relative light units (RLU) min^−1^ mg of aorta^−1^.

### Flow cytometry

Aortae, MLNs, spleen and blood were collected from all the animals. Next, the aortae were digested with aorta dissociation enzyme stock solution (activity units (U)/ml): collagenase type XI 125, hyaluronidase type 1 60, collagenase type 1 450, and DNase I 60 (Merck, Darmstadt, Germany) at 37 °C for 1 h. The samples were adequately homogenized with wet slides to reduce friction, and then, the solutions were filtered through a 40 µM cell strainer. Red blood cells from the blood and spleen were lysed with red blood cell lysis buffer (BioLegend, San Diego, USA).

Next, the samples were pipetted into polystyrene tubes for staining. All the samples were first incubated with a viability dye (LIVE/DEAD® Fixable Aqua Dead Cell Stain Kit, Molecular Probes, Oregon, USA.) in PBS for 20 min at 4 °C in the dark. Next, the cells were stained for extracellular and intracellular markers in sequential incubations at 4 °C. Additionally, the samples were permeabilized and fixed. The list of antibodies and their targets used can be found in Table S2.

Samples were processed with a flow cytometer, FACS Symphony A5 SORP BD (BD Biosciences), and analysis was carried out with FlowJo software and a gating strategy, as illustrated in Figure S13.

### Gene and protein expression analysis

As reported previously,^11^ gene expression analysis was performed by quantitative polymerase chain reaction (qPCR). Total RNA from the colon and PVN was extracted with PRImeZOL™ Reagent (Canvax, Valladolid, Spain). The RNA concentrations were calculated using a NanoDrop™ 2000 Spectrophotometer (Thermo Fisher Scientific, Inc., Waltham, MA, USA), and 1 µg was retrotranscribed to cDNA with an iScript™ cDNA synthesis kit (Bio-Rad, California, USA). qPCR was performed with a CFX Duet Real-Time PCR System (Bio-Rad, California, USA). The sequence primers used for amplification are listed in Table S3. The mRNA expression fold change was normalized to that of the control sample and processed by the ΔΔCt method, using glyceraldehyde-3-phosphate dehydrogenase (*Gadph*) used as a housekeeping gene.

We assessed protein expression via western blotting according to our previous reports.^63^ Proteins were isolated from colon samples and quantified via a BCA protein assay. To separate proteins by molecular weight, 20 µg of protein was run in a sodium‒dodecyl sulfate‒polyacrylamide mixture and then transferred to polyvinylidene difluoride membranes. Membranes were blocked with 5% BSA in Tris-buffered saline with Tween 20 solution (TBS-T, 0.5 M Tris pH 7.5; 1.5 M NaCl; 0.1% Tween 20) during 1 h at room temperature.

Next, membranes were incubated with primary antibodies overnight at 4 °C, ZO-1 and occludin were detected: rabbit polyclonal anti-occludin (1:1000, Abcam, Cambridge, USA Cat# ab216327, RRID:AB_2737295) and rabbit polyclonal anti-tight junction protein ZO-1 (1:1000; Invitrogen, Carlsbad, CA Cat# 61-7300, RRID:AB_2533938). All the antibodies were diluted in TBS-T with 3% BSA and 0.001% azide. The membranes were then exposed with secondary peroxidase-conjugated goat anti-rabbit antibody (1:10000; Santa Cruz Biotechnology, Texas, USA.) for 1 h at room temperature. In order to detect antibody binding, an electrochemiluminescence (ECL) system (Amersham Pharmacia Biotech, Amersham, UK) was used. Densitometric analysis of the resulting bands was carried out via ImageJ software, and the results were normalized to the *β*-actin bands.

### DNA extraction, 16S rRNA gene amplification, bioinformatics

We collected fecal samples at the experimental endpoint for intestinal microbial population analysis. The protocol followed is described in detail in our previous publication.^11^ DNA was extracted using G-spin columns (INTRON Biotechnology). The V4–V5 regions of the 16S rRNA gene were then amplified from all the samples. Amplicon libraries were generated using a Bioanalyzer 2100 (Agilent). Sequencing was performed at the Unidad de Genómica (Parque Científico de Madrid, Spain) on an Illumina MiSeq instrument using paired-end 2 × 300 bp sequencing. High-quality 16S rRNA amplicon sequences were analyzed via TADA (Targeted Amplicon Diversity Analysis using DADA2, implemented in NextFlow. The data set for mapping is from Greengenes2,^64^ with forward and reverse truncate lengths 285/250 bp and a maximum expected error of 2. The Microbiome Analyst pipeline (REF) was used for downstream analysis. Metagenomic prediction was performed using Phylogenetic Investigation of Communities by Reconstruction of Unobserved States (PICRUSt) according to Kyoto Encyclopedia of Genes and Genomes (KEGG) pathways. The data are presented as the relative abundance of predicted functions within the samples. The abundance of KEGG modules was calculated by summing the abundance of genes annotated to the same feature. The pathways of interest were also presented as the relative abundance of predicted functions within the samples.^50^ Bacterial genera were classified according to their predominant SCFA production, acetate, propionate, or butyrate, based on previously established criteria.^42^

### Histological evaluation of gut pathologies and immunohistochemistry staining in brain

For histological studies, rat colon tissues were isolated from all groups, immediately fixed in buffered 10% formaldehyde, paraffin-embedded, and transverse sections were stained with hematoxylin‒eosin and Masson's trichrome stain. Histochemical staining was interpreted and scored simultaneously by two independent investigators (F.O. and N.M-M.). The histological analysis was performed in a blinded fashion on 4-micrometer sections via light microscopy. Histopathological changes in the colon were assessed by evaluating morphology (percentage of goblet cells in glandular crypts). ImageJ software v1.48 (http://imagej.nih.gov/ij/) was used to assess the area occupied by muscle, connective tissue, and microvessels, and the length of the glandular crypts was measured in triplicate in each image. A total of 120 images (20× magnification) were taken with a CD70 camera coupled to a BX51 microscope (Olympus Optical Company, Ltd., Tokyo, Japan).

For immunohistochemistry (IHC), paraffin-embedded coronal sections of the rat nervous system were deparaffinized, hydrated, and heat-treated in 1 mM EDTA (pH 8) for antigenic unmasking in an antigen retrieval PT module (Thermo Fisher Scientific Inc., Waltham, MA, USA) at 95 °C for 20 min. The sections were incubated for 1 h at room temperature with concentrated polyclonal anti-Iba1 (1:100) (Invitrogen™, Thermo Fisher Scientific, Langenselbold, Germany), a marker expressed at the cell membrane and cytoplasm by microglia. An immunocytochemical study was conducted using the micropolymer-peroxidase-based method (Master Polymer, Vitro-Master Diagnóstica, Granada, Spain), followed by development with diaminobenzidine. Mayer's hematoxylin was used for nuclear counterstaining. Appropriate positive and negative (nonimmune serum) controls were used concurrently. The IHC study was performed in a blinded fashion on 4 μm sections via light microscopy on a millimeter scale under a microscope (BH2, Olympus Optical Company, Ltd.) with a 40× objective. The number of positive cells was quantified per mm^2^.

### Plasma determinations

Plasma was collected from blood by centrifuging for 10 min at 3500  rpm and 4 °C. The resulting plasma was aliquoted and stored at −80 °C until use. I-FABP2, NA, and LPS concentrations in plasma were measured through commercially available kits (the I-FABP ELISA Kit, R& D Systems, Minneapolis, MN; the noradrenaline ELISA Kit, IBL International, Hamburg, Germany; and the Amebocyte Lysate Chromogenic Endotoxin Quantitative Kit, Lonza, Valais, Switzerland), following the manufacturer's protocols. Concentrations of SCFAs in rat plasma were measured in duplicate through gas chromatography, employing a previously reported methodology.^23^ The method demonstrated acceptable precision across a range of concentrations, with coefficients of variation ranging from 4.9% to 9.5%.

HCTZ levels in plasma were determined using Cortecs UPLC C18 column (2.1 × 100 mm, 1.6 µm) coupled to an Acquity UPLC System I-Class chromatograph (Waters Corporation, Cerdanyola del Vallès, Spain). The mobile phase containing 0.1% NH_3_ in water (A) and methanol (B) was at a flow rate of 0.3 mL min^−1^. The gradient program was as follows: 20% B, T5: 95% B, T5.1: 20% B. The separation temperature was set at 50 °C, and the sample injection volume was 4 µL. MS detection was performed on a Xevo TQ-XS Triple Quadrupole mass spectrometer (Waters Corporation, Cerdanyola del Vallès, Spain) coupled with an electrospray ionization (ESI) source. The MS instrument was set in both positive and negative ion modes. Calibration curves for HCTZ were prepared using the internal standard method. HCTZ was diluted in working solution to reach different concentrations of the curve. The samples were then centrifuged at 16000 × *g* for 10 min to obtain clean supernatants. Finally, 4 µL of supernatant was injected into the UPLC‒MS system for analysis.^65^^,^^66^

### Reagents

Unless otherwise noted, the chemicals and other reagents were purchased from Merck (Barcelona, Spain).

### Statistical analysis

Reads per OTU were normalized to total reads in each sample. Prior to statistical analysis, the sequencing data were subjected to a comprehensive filtering and normalization procedure. Initially, two-step low-count filtering was implemented to minimize sequencing artifacts and low-level contamination. Features with fewer than 4 counts and those present in less than 20% of the samples were excluded. A low-variance filter based on the interquartile range (IQR) was subsequently applied to remove features with variability below the 10th percentile. Following these filtering steps, data normalization was performed using rarefaction curves to account for differences in sequencing depth. The resulting data were then scaled using Total Sum Scaling (TSS) to ensure comparability across samples. The phyloseq package was used to calculate alpha diversity parameters. Taxonomic differences between experimental groups were assessed using Partial Least Square Discriminant Analysis (PLS-DA). To address the issue of multiple comparisons and reduce the risk of false-positive results in microbiome and gene expression analyses, we applied false discovery rate (FDR) correction using the Benjamini‒Hochberg procedure. Adjusted *p* values are reported where appropriate.

For physiological, biochemical, and vascular data, statistical analyses were performed using Prism V8 (GraphPad Software Inc., USA). When two groups were compared, an unpaired *t*-test was performed. Tail cuff SBP determination and vascular reactivity results were analyzed via two-way repeated-measures ANOVA with Sidak's multiple comparison test. Data normality was assessed using the Shapiro‒Wilk normality test. When the data followed a normal distribution, comparisons among multiple groups were performed using one-way ANOVA with Tukey's post hoc test. For nonnormally distributed data, the Mann‒Whitney U test (for two groups) or the Kruskal‒Wallis test with Dunn's multiple comparison test (for more than two groups) was used. A *p* value < 0.05 was considered statistically significant.

## Acknowledgments

The authors thank *N*. Rodríguez for technical assistance.

## Author contributors

JD is responsible for the overall content as the guarantor. JD conceived the project, designed the experiments, analyzed the data, and wrote the manuscript. JM, CG-C, SM, MT, IRV, MS, RJ, MO, MR, and MG-G designed and performed most of the experiments and analyzed the data. NM-M and FO performed the histological methodology, and EG-H performed the SCFA determinations. FO assisted with data analysis and interpretation and critically read the manuscript.

## Supplementary Material

Supplementary material Supplementary Material.

Supplementary material Supplementary figures and tables.

## Data Availability

The sequencing dataset from this study has been deposited in ZENODO (Doi: 10.5281/zenodo.15081781).
