# Supplementary material for: The probiotic Limosilactobacillus fermentum CECT5716 enhances the antihypertensive response to hydrochlorothiazide in spontaneously hypertensive rats
Source: Gut Microbes. 2025 Nov 18;17(1):2586324. doi: 10.1080/19490976.2025.2586324 (PMC12645898; doi:10.1080/19490976.2025.2586324)

Fig 5C

ZO-1 WB

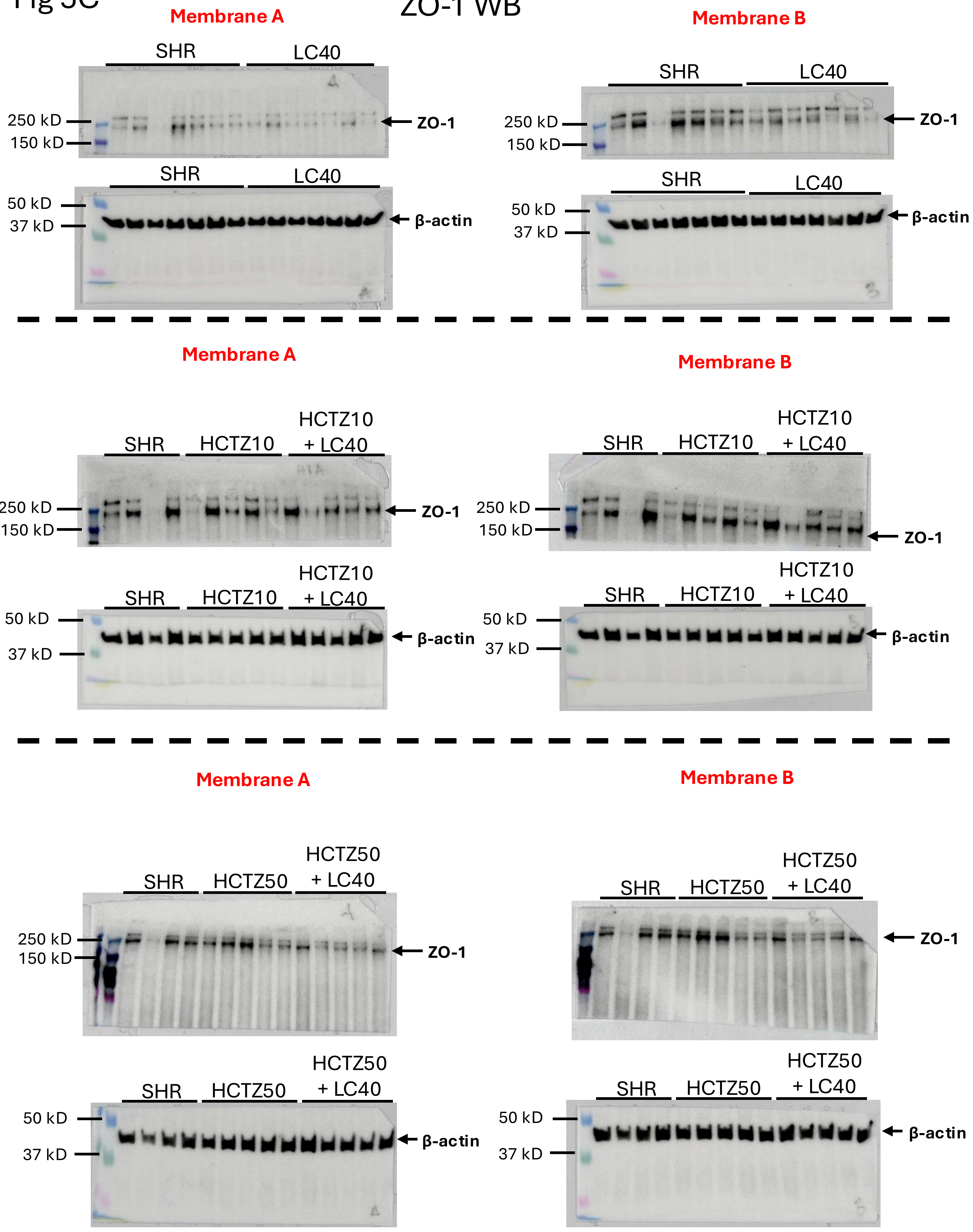

Membrane A
SHR
LC40
250 kD
150 kD
ZO-1
SHR
LC40
50 kD
37 kD
β-actin
Membrane B
SHR
LC40
250 kD
150 kD
ZO-1
SHR
LC40
50 kD
37 kD
β-actin
Membrane A
SHR
HCTZ10
HCTZ10 + LC40
250 kD
150 kD
ZO-1
SHR
HCTZ10
HCTZ10 + LC40
50 kD
37 kD
β-actin
Membrane B
SHR
HCTZ10
HCTZ10 + LC40
250 kD
150 kD
ZO-1
SHR
HCTZ10
HCTZ10 + LC40
50 kD
37 kD
β-actin
Membrane A
SHR
HCTZ50
HCTZ50 + LC40
250 kD
150 kD
ZO-1
SHR
HCTZ50
HCTZ50 + LC40
50 kD
37 kD
β-actin
Membrane B
SHR
HCTZ50
HCTZ50 + LC40
ZO-1
SHR
HCTZ50
HCTZ50 + LC40
50 kD
37 kD
β-actin

Fig 5C

Occludin WB

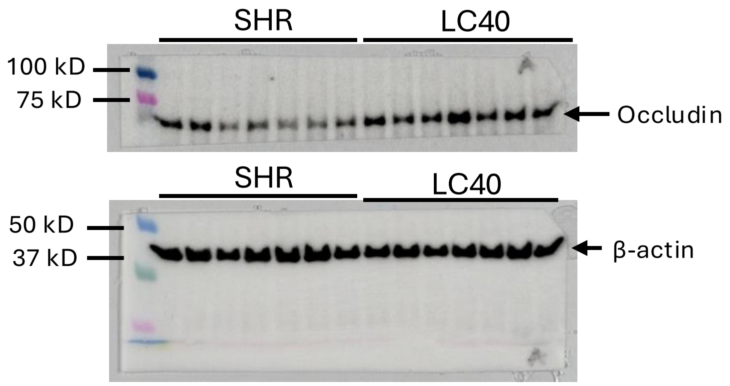

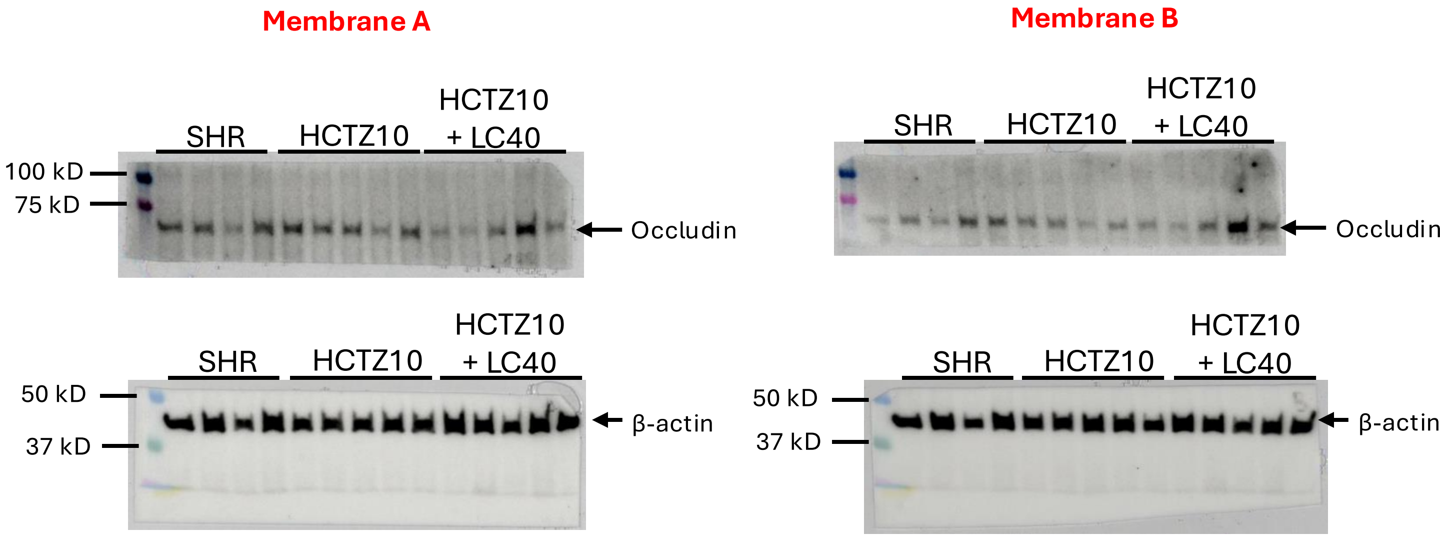

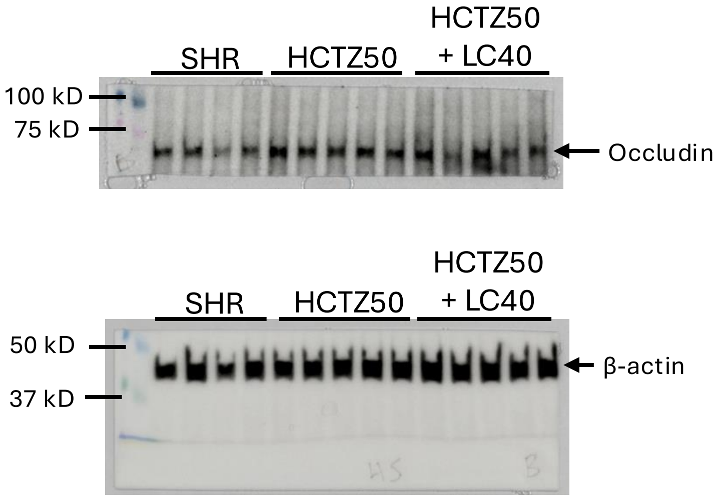

Fig 5C

β-actina

Tiempo de exposición: 1s

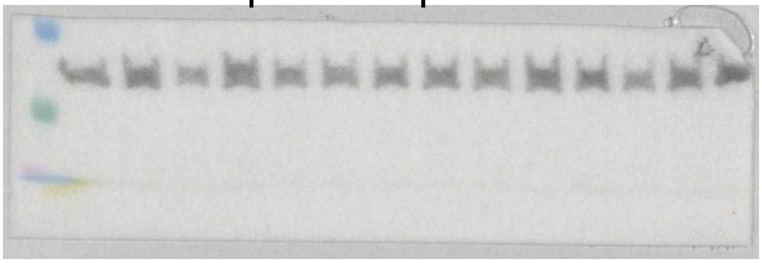

Tiempo de exposición: 10s

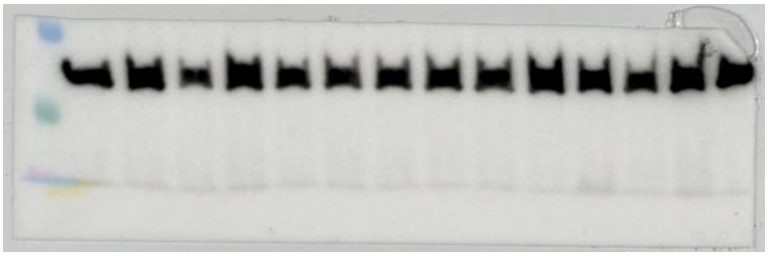

Supplement: Supplementary material [file KGMI_A_2586324_SM1570.pdf]
