# Supplementary material for: The probiotic Limosilactobacillus fermentum CECT5716 enhances the antihypertensive response to hydrochlorothiazide in spontaneously hypertensive rats
Source: Gut Microbes. 2025 Nov 18;17(1):2586324. doi: 10.1080/19490976.2025.2586324 (PMC12645898; doi:10.1080/19490976.2025.2586324)
Supplement: Supplementary material — Supplementary figures and tables. [file KGMI_A_2586324_SM1569.docx]

**Title: The probiotic *Limosilactobacillus fermentum* CECT5716 enhances the antihypertensive response to hydrochlorothiazide in spontaneously hypertensive rats**

González-Correa, C^a,b,*^, Miñano, S^a,b,*^, Moleón, J^a,b,c,*^, Toral, M^a,b,d^, Robles-Vera, I^e^, Sánchez, M^a,b^, Jiménez, R^a,b,d^, Olivares, M^f^, Martín-Morales N^g^, O’Valle F^b,g^, Guerra-Hernández, E^h^, Romero, M^a,b^, Gómez-Guzmán, M^a,b^, Duarte, J^a,b,d^

**Figure S1. Chronic hydrochlorothiazide (HCTZ) treatment had an inhibitory effect on vascular contractility which was not affected by *Limosilactobacillus fermentum* CECT5716 (LC40) administration.** Concentration-contractile response curve induced by phenylephrine (Phe). Values are expressed as mean ± SEM (n = 8). Groups: untreated spontaneously hypertensive rats (SHR, vehicle (methylcellulose 1%) 1 mL day^-1^) group, treated group with 10^9^ CFU day^-1^ LC40 (LC40), treated group with 10 mg Kg^-1^ day^-1^ HCTZ (HCTZ10), treated group with 50 mg Kg^-1^ day^-1^ HCTZ (HCTZ50), co-treated group with LC40 and HCTZ (HCTZ10+LC40) and co-treated group with LC40 and HCTZ (HCTZ50+LC40). Data were analysed by two-way repeated-measures ANOVA with Tukey’s multiple comparison test. *P<0.05 and **P<0.001 significant differences compared with SHR group.

**Figure S2. Microbiota rarefaction curves were obtained across all experimental groups.** Groups: untreated spontaneously hypertensive rats (SHR, vehicle (methylcellulose 1%) 1 mL day^-1^) group, treated group with 10^9^ CFU day^-1^ LC40 (LC40), treated group with 10 mg Kg^-1^ day^-1^ HCTZ (HCTZ10), treated group with 50 mg Kg^-1^ day^-1^ HCTZ (HCTZ50), co-treated group with LC40 and HCTZ (HCTZ10+LC40) and co-treated group with LC40 and HCTZ (HCTZ50+LC40).

**Figure S3. *Limosilactobacillus fermentum* CECT5716 (LC40), hydrochlorothiazide (HCTZ) and LC40 co-administration with HCTZ change gut microbiota at family levels. (A)** Families proportion in the fecal samples and **(B)** families that have more than 1% relative abundance of total bacteria and show statistical differences between experimental groups. Values are expressed as mean ± SEM (n =6-8). Groups: untreated group spontaneously hypertensive rats (SHR, vehicle (methylcellulose 1%) 1 mL day^-1^), treated group with 10^9^ CFU day^-1^ LC40 (LC40), treated group with 10 mg Kg^-1^ day^-1^ HCTZ (HCTZ10), treated group with 50 mg Kg^-1^ day^-1^ HCTZ (HCTZ50), co-treated group with LC40 and HCTZ (HCTZ10+LC40) and co-treated group with LC40 and HCTZ (HCTZ50+LC40). Data were analyzed by one-way ANOVA with a Tukey’s post-hoc test. *P<0.05 and **P<0.01 significant differences compared with SHR group. ^#^P<0.05 significant differences compared with HCTZ10 group.

**Figure S4.** ***Limosilactobacillus fermentum* CECT5716 (LC40), hydrochlorothiazide (HCTZ) and LC40 co-administration with HCTZ change gut microbiota at genus levels. (A)** Heat map of bacterial genus. The heatmap colors represent the relative percentage of microbial genera assigned within each sample. **(B)** Relative abundance of main bacterial genus in the different experimental groups. Values are expressed as mean ± SEM (n = 6-8). Groups: untreated group spontaneously hypertensive rats (SHR, vehicle (methylcellulose 1%) 1 mL day^-1^), treated group with 10^9^ CFU day^-1^ LC40 (LC40), treated group with 10 mg Kg^-1^ day^-1^ HCTZ (HCTZ10), treated group with 50 mg Kg^-1^ day^-1^ HCTZ (HCTZ50), co-treated group with LC40 and HCTZ (HCTZ10+LC40) and co-treated group with LC40 and HCTZ (HCTZ50+LC40). Data were analyzed by one-way ANOVA with a Tukey’s post-hoc test. *P<0.05, **P<0.01 and ***P<0.001 significant differences compared with SHR group. ^#^P<0.05 significant differences compared with HCTZ10 group.

**Figure S5. *Limosilactobacillus fermentum* CECT5716 (LC40), hydrochlorothiazide (HCTZ) and LC40 co-administration with HCTZ change gut microbial gene functions. (A)** The average abundance of KEGG modules changed by HCTZ in spontaneously hypertensive rats (SHR) gut microbiome. **(B)** Genes abundance related to lipopolysaccharide biosynthesis and export system in all experimental groups. Values are expressed as mean ± SEM (n = 6-8). Groups: untreated group SHR (vehicle (methylcellulose 1%) 1 mL day^-1^), treated group with 10^9^ CFU day^-1^ LC40 (LC40), treated group with 10 mg Kg^-1^ day^-1^ HCTZ (HCTZ10), treated group with 50 mg Kg^-1^ day^-1^ HCTZ (HCTZ50), co-treated group with LC40 and HCTZ (HCTZ10+LC40) and co-treated group with LC40 and HCTZ (HCTZ50+LC40). Data were analyzed by one-way ANOVA with a Tukey’s post-hoc test. *P<0.05, **P<0.01 and ***P<0.001 significant differences compared with SHR group. ^#^P<0.05 significant differences compared with HCTZ10 group.

**Figure S6. *Limosilactobacillus fermentum* CECT5716 (LC40) and co-administration with hydrochlorothiazide (HCTZ) improves T cell profile at Mesenteric Lymph Nodes (MLNs) and spleen in spontaneously hypertensive rats (SHR).** T helper (Th)-17, regulatory T cells (Treg) and Th1, gate on CD3+CD4+ cells, measured by flow cytometry in MLNs and spleen. Values are expressed as mean ± SEM (n = 7-8). Groups: untreated group SHR (vehicle (methylcellulose 1%) 1 mL day^-1^), treated group with 10^9^ CFU day^-1^ LC40 (LC40), treated group with 10 mg Kg^-1^ day^-1^ HCTZ (HCTZ10), treated group with 50 mg Kg^-1^ day^-1^ HCTZ (HCTZ50), co-treated group with LC40 and HCTZ (HCTZ10+LC40) and co-treated group with LC40 and HCTZ (HCTZ50+LC40). Data were analyzed by one-way ANOVA with a Tukey’s post-hoc test. *P<0.05, **P<0.01 and ***P<0.001 significant differences compared with SHR group. ^#^P<0.05 and ^##^P<0.01 significant differences compared with HCTZ groups.

**Figure S7. Rarefaction curves of the microbiota following fecal microbiota transplantation from untreated and treated spontaneously hypertensive rat (SHR) donors to Wistar Kyoto (WKY) recipients.** Groups: WKY with SHR microbiota (W-SHR), WKY with microbiota from *Limosilactobacillus fermentum* CECT5716 **(**LC40) group (W-LC40), WKY with microbiota from hydrochlorothiazide **(**HCTZ)10 group (W-HCTZ10), WKY with microbiota from HCTZ50 group (W-HCTZ50), WKY with microbiota from HCTZ10+LC40 group (W-HCTZ10+LC40), and WKY with HCTZ50+LC40 group microbiota (W- HCTZ50+LC40).

**Figure S8. Microbiota composition following fecal microbiota transplantation from untreated and treated spontaneously hypertensive rat (SHR) donors to Wistar Kyoto (WKY) recipients. (A)** The microbial DNA from fecal samples was analyzed by 16S rRNA gene sequencing. Ecological parameters of richness, such as Chao, and abundance-based coverage estimator (ACE), and diversity, such as Shannon and Simpson. **(B)** Principal coordinate analysis (PCoA) in the gut microbiota from all experimental groups. **(C)** Phylum breakdown of the seven most abundant bacterial communities in the fecal samples was obtained from all experimental groups. **(D)** Firmicutes/Bacteroidota (F/B) ratio was calculated as a biomarker of gut dysbiosis. **(E)** Relative proportion of total acetate-, total butyrate- and total propionate-producing bacteria expressed as relative abundance of total bacteria. Values are expressed as mean ± SEM (n = 7-8). Groups: WKY with SHR microbiota (W-SHR), WKY with microbiota from *Limosilactobacillus fermentum* CECT5716 **(**LC40) group (W-LC40), WKY with microbiota from hydrochlorothiazide **(**HCTZ)10 group (W-HCTZ10), WKY with microbiota from HCTZ50 group (W-HCTZ50), WKY with microbiota from HCTZ10+LC40 group (W-HCTZ10+LC40), and WKY with HCTZ50+LC40 group microbiota (W-HCTZ50+LC40). Data were analyzed by one-way ANOVA with a Tukey’s post-hoc test. *P<0.05 significant differences compared with W-SHR group. ^#^P<0.05 significant differences compared with W-HCTZ50 group.

**Figure S9.** **Effects of fecal microbiota transplantation from untreated and treated spontaneously hypertensive rat (SHR) donors to normotensive Wistar Kyoto (WKY) recipients on microbiota composition at the genus level. (A)** Heat map of bacterial genus. The heatmap colors represent the relative percentage of microbial genera assigned within each sample. **(B)** Relative abundance of bacterial main genus with a relative abundance > 1% in the different experimental groups. Values are expressed as mean ± SEM (n = 7-8). Groups: WKY with SHR microbiota (W-SHR), WKY with microbiota from *Limosilactobacillus fermentum* CECT5716 **(**LC40) group (W-LC40), WKY with microbiota from hydrochlorothiazide **(**HCTZ)10 group (W-HCTZ10), WKY with microbiota from HCTZ50 group (W-HCTZ50), WKY with microbiota from HCTZ10+LC40 group (W-HCTZ10+LC40), and WKY with HCTZ50+LC40 group microbiota (W-HCTZ50+LC40). Data were analyzed by one-way ANOVA with a Tukey’s post-hoc test. *P<0.05significant differences compared with W-SHR group. ^#^P<0.05 significant differences compared with HCTZ groups.

**Figure S10. Effects of fecal microbiota transplantation (FMT) from treated spontaneously hypertensive rats (SHR) to normotensive Wistar Kyoto rats (WKY) on aortic contractility.** Concentration-contractile response curve induced by phenylephrine (Phe). Values are expressed as mean ± SEM (n = 8). Groups: FMT from SHR to WKY (W-SHR), from LC40 to WKY rats (W-LC40), from HCTZ10 group to WKY rats (W- HCTZ10), from HCTZ10 + LC40 group to WKY rats (W-HCTZ10+LC40), from HCTZ50 group to WKY rats (W- HCTZ50) and from HCTZ50 + LC40 group to WKY rats (W-HCTZ50+LC40). Data were analysed by two-way repeated-measures ANOVA with Tukey’s multiple comparison test.

**Figure S11. Inhibition of NADPH oxidase activity improved endothelial dysfunction induced by fecal microbiota transplantation (FMT) from untreated spontaneously hypertensive rats (SHR) to normotensive Wistar Kyoto rats (WKY).** Endothelium-dependent relaxation induced by acetylcholine (ACh) in aortas precontracted with phenylephrine (Phe), in presence of the selective NADPH oxidase inhibitor VAS2870. Values are expressed as mean ± SEM (n = 8). Groups: FMT from SHR to WKY (W-SHR), from LC40 to WKY rats (W-LC40), from HCTZ10 group to WKY rats (W- HCTZ10), from HCTZ10 + LC40 group to WKY rats (W-HCTZ10+LC40), from HCTZ50 group to WKY rats (W- HCTZ50) and from HCTZ50 + LC40 group to WKY rats (W-HCTZ50+LC40). Data were analysed by two-way repeated-measures ANOVA with Tukey’s multiple comparison test.

**Figure S12. Protective effects of aortic regulatory T cell (Treg) infiltration induced by fecal microbiota transplantation (FMT) from treated spontaneously hypertensive rats (SHR) on endothelial dysfunction caused by FMT from untreated SHR to normotensive Wistar Kyoto rats (WKY). (A)** Relative proportion of Tregs, gate on CD3+CD4+ cells, measured by flow cytometry in mesenteric lymph nodes, spleen and aorta. **(B)** Endothelium-dependent relaxation induced by acetylcholine (ACh) in aortas precontracted with phenylephrine (Phe), after 3 h of incubation with physiological buffer solution (PBS) or neutralizing interleukin-10 antibody (nIL-10). Values are expressed as mean ± SEM (n = 4). Groups: FMT from SHR to WKY (W-SHR), from LC40 to WKY rats (W-LC40), from HCTZ10 group to WKY rats (W- HCTZ10), from HCTZ10 + LC40 group to WKY rats (W-HCTZ10+LC40), from HCTZ50 group to WKY rats (W- HCTZ50) and from HCTZ50 + LC40 group to WKY rats (W-HCTZ50+LC40). Treg abundance data were analyzed by one-way ANOVA with a Tukey’s post-hoc test. ACh relaxation data were analysed by two-way repeated-measures ANOVA with Tukey’s multiple comparison test.

**Figure S13. Gating strategy for flow cytometry.**

**Table S1.** Effect of *Lactobacillus fermentum* (LC40), and hydrochlorothiazide (HCTZ) on phyla proportion (%) of gut microbiota from spontaneously hypertensive rats (SHR).

|  | **SHR**  (n = 6) | **LC40**  (n = 7) | P value (LC40 *vs* SHR) |  | **HCTZ10**  (n = 7) | P value (*vs* SHR) |  | **HCTZ10+LC40**  (n = 8) | P value (*vs* HCTZ10) | **HCTZ50**  (n = 8) | P value (*vs* SHR) | **HCTZ50+LC40**  (n = 8) | P value (*vs* HCTZ50) |
| --- | --- | --- | --- | --- | --- | --- | --- | --- | --- | --- | --- | --- | --- |
| **Actinobacteriota** | 0.01 ± 0.01 | 0.04 ± 0.02 | 0.184 |  | 0.01 ± 0.01 | 0.627 |  | 0.01 ± 0.01 | 0.581 | 0.07 ± 0.05 | 0.356 | 0.08 ± 0.05 | 0.863 |
| **Bacteroidota** | 22.48 ± 6.02 | 39.20 ± 5.91 | 0.055 |  | 20.71 ± 6.21 | 0.829 |  | 37.79 ± 4.65 | 0.032 | 42.46 ± 5.17 | 0.019 | 46.19 ± 3.66 | 0.540 |
| **Cyanobacteria** | 0.05 ± 0.02 | 0.19 ± 0.09 | 0.160 |  | 0.08 ± 0.07 | 0.592 |  | 0.15 ± 0.06 | 0.467 | 0.17 ± 0.06 | 0.113 | 0.71 ± 0.30 | 0.077 |
| **Desulfobacterota** | 0.72 ± 0.22 | 1.66 ± 1.37 | 0.509 |  | 0.59 ± 0.15 | 0.598 |  | 0.58 ± 0.24 | 0.969 | 0.47 ± 0.35 | 0.568 | 0.41 ± 0.13 | 0.865 |
| **Firmicutes** | 76.36 ± 6.11 | 57.87 ± 7.19 | 0.061 |  | 78.22 ± 6.32 | 0.824 |  | 60.43 ± 4.69 | 0.028 | 55.46 ± 5.24 | 0.016 | 51.37 ± 4.11 | 0.523 |
| **Proteobacteria** | 0.35 ± 0.17 | 0.86 ± 0.19 | 0.054 |  | 0.37 ± 0.19 | 0.919 |  | 1.02 ± 0.26 | 0.051 | 1.28 ± 0.29 | 0.017 | 1.00 ± 0.23 | 0.425 |
| **Verrucomicrobiota** | 0.03 ± 0.02 | 0.18 ± 0.13 | 0.308 |  | 0.10 ± 0.05 | 0.283 |  | 0.01 ± 0.00 | 0.464 | 0.09 ± 0.05 | 0.346 | 0.23 ± 0.10 | 0.211 |

An unpaired t-test was used to compare differences between two groups

**Table S2. Antibodies list for flow cytometry**

| **Antibody** | **Clone** | **Source** | **Identifier** |
| --- | --- | --- | --- |
| LIVE/DEAD AmCyan |  | Invitrogen | L34957 |
| CD45 FITC | REA504 | Miltenyi | 130-107-794 |
| CD3 APC | REA223 | Miltenyi | 130-102-679 |
| CD4 PerCP-Vio700 | REA489 | Miltenyi | 130-107-505 |
| IFNγ PE | DB-1 | BioLegend | 507806 |
| FoxP3 PE-Cyanine7 | FJK-16s | eBioscience | 25-5773-82 |
| IL17a BV421 | eBio17B7 | eBioscience | 404-7177-82 |

**Table S3. Oligonucleotides for real-time RT-PCR**

| **mRNA targets** | **Descriptions** | **Sense** | **Antisense** |
| --- | --- | --- | --- |
| *Il-1β* | Interleukin-1 beta | GTCACTCATTGTGGCTGTGG | GCAGTGCAGCTGTCTAATGG |
| *Occludin* | Occludin | ACACAGACCCCAGAGCGGCA | AGCCTGGGCAGTCGGGTTGA |
| *Zo-1* | Zonula occludens 1 | GCCAGCCAGTTCCGCCTCTG | AGGGTCCTCCCGGGTTGGTG |
| *Muc-2* | Mucin 2 | ACCACCATTACCACCACCTCAG | CGATCACCACCATTGCCACTG |
| *Muc-3* | Mucin 3 | CACAAAGGCAAGAGTCCAGA | ACTGTCCTTGGTGCTGCTGAATG |
| *IFNγ* | Interferon gamma | GCCCTCTCTGGCTGTTACTG | CCAAGAGGAGGCTCTTTCCT |
| *Nox-1* | NOX-1 subunit of NADPH oxidase | TCTTGCTGGTTGACACTTGC | TATGGGAGTGGGAATCTTGG |
| *Ccl2* | C-C chemokine ligand 2 | CCTCCACCACTATGCAGGTC | CAGCCGACTCATTGGGATCA |
| *Cd11b* | CD11b | GAGAACTGGTTCTGGCTTGC | TCAGTTCGAGCCTTCTT |
| *Tlr4* | Toll-like receptor 4 | AGACCAGGAAGCTTGAATCCCTGC | GCCATGCCATGCCTTGTCTTCA |
| *p22^phox^* | p22phox subunit of NADPH oxidase | GCGGTGTGGACAGAAGTACC | CTTGGGTTTAGGCTCAATGG |
| *Th* | Tyrosine hydroxylase | GATTGCTACCTGGAAGGAGGT | AGTCCAATGTCCTGGGAGAAC |
| *Gadph* | Glyceraldehyde-3-phosphate dehydrogenase | GTCGGTGTGAACGGATTT | ATGGGTTTCCCGTTGATG |
